# Outcomes of Minimally Invasive Thyroid Surgery – A Systematic Review and Meta-Analysis

**DOI:** 10.3389/fendo.2021.719397

**Published:** 2021-08-12

**Authors:** Lisa H. de Vries, Dilay Aykan, Lutske Lodewijk, Johanna A. A. Damen, Inne H. M. Borel Rinkes, Menno R. Vriens

**Affiliations:** ^1^Department of Surgical Oncology and Endocrine Surgery, University Medical Center Utrecht, Utrecht, Netherlands; ^2^Julius Center for Health Sciences and Primary Care, University Medical Center Utrecht, Utrecht University, Utrecht, Netherlands

**Keywords:** minimally invasive video assisted thyroidectomy (MIVAT), bilateral axillo-breast approach endoscopic thyroidectomy (BABA-ET), bilateral axillo-breast approach robotic thyroidectomy (BABA-RT), transoral endoscopic thyroidectomy *via* vestibular approach (TOETVA), retro-auricular endoscopic thyroidectomy (RA-ET), retro-auricular robotic thyroidectomy (RA-RT), gasless transaxillary endoscopic thyroidectomy (GTET), robot assisted transaxillary surgery (RATS)

## Abstract

**Purpose:**

Conventional thyroidectomy has been standard of care for surgical thyroid nodules. For cosmetic purposes different minimally invasive and remote-access surgical approaches have been developed. At present, the most used robotic and endoscopic thyroidectomy approaches are minimally invasive video assisted thyroidectomy (MIVAT), bilateral axillo-breast approach endoscopic thyroidectomy (BABA-ET), bilateral axillo-breast approach robotic thyroidectomy (BABA-RT), transoral endoscopic thyroidectomy *via* vestibular approach (TOETVA), retro-auricular endoscopic thyroidectomy (RA-ET), retro-auricular robotic thyroidectomy (RA-RT), gasless transaxillary endoscopic thyroidectomy (GTET) and robot assisted transaxillary surgery (RATS). The purpose of this systematic review was to evaluate whether minimally invasive techniques are not inferior to conventional thyroidectomy.

**Methods:**

A systematic search was conducted in Medline, Embase and Web of Science to identify original articles investigating operating time, length of hospital stay and complication rates regarding recurrent laryngeal nerve injury and hypocalcemia, of the different minimally invasive techniques.

**Results:**

Out of 569 identified manuscripts, 98 studies met the inclusion criteria. Most studies were retrospective in nature. The results of the systematic review varied. Thirty-one articles were included in the meta-analysis. Compared to the standard of care, the meta-analysis showed no significant difference in length of hospital stay, except a longer stay after BABA-ET. No significant difference in incidence of recurrent laryngeal nerve injury and hypocalcemia was seen. As expected, operating time was significantly longer for most minimally invasive techniques.

**Conclusions:**

This is the first comprehensive systematic review and meta-analysis comparing the eight most commonly used minimally invasive thyroid surgeries individually with standard of care. It can be concluded that minimally invasive techniques do not lead to more complications or longer hospital stay and are, therefore, not inferior to conventional thyroidectomy.

## Introduction

During the last century the number of detected thyroid nodules has increased significantly. At present, nearly 5% of the female population has a thyroid nodule (1). For thyroid nodules that require surgery, conventional thyroidectomy has been standard of care (2). The extent of surgery varies from lobectomy to total thyroidectomy. Due to new diagnostic techniques to detect small malignant lesions and better surveillance, the number of thyroidectomies performed has increased substantially (3). Conventional thyroidectomy provides excellent exposure of the thyroid but leaves the patient with a visible scar in the lower region of the neck. Since most patients with a thyroid nodule are young women, different techniques have emerged to avoid a potentially disfiguring scar (2).

**Figure 1:**
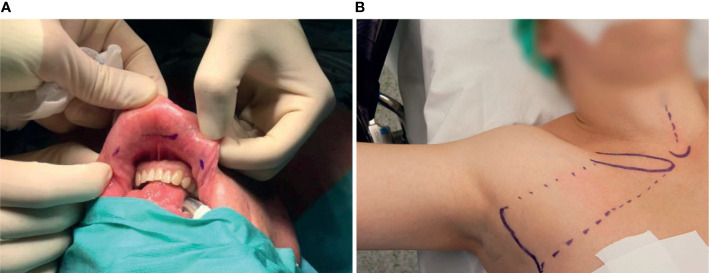
Preoperatively marked patients who will undergo **(A)** TOETVA (7). Three incisions will be made in the oral vestibule, one for the camera and two laterally for dissection and coagulation instruments; and **(B)** RATS (8). The incision site is marked laterally. The dotted lines mark the borders of the surgical working space.

The first innovative thyroid technique was the endoscopic thyroidectomy, first performed in 1997 by Huscher (4). Two years later Miccoli performed the first minimally invasive video assisted thyroidectomy (MIVAT) (5). In the following years different types of endoscopic remote access techniques were introduced. Of the many endoscopic remote access techniques, the gasless transaxillary endoscopic thyroidectomy (GTET), bilateral axillo-breast approach (BABA), retro-auricular (RA) facelift approach, and transoral endoscopic thyroidectomy *via* vestibular approach (TOETVA) are the most commonly used techniques today (6). To date TOETVA, as shown in **Figure 1**, is the only technique performed without cutaneous scars (9). In 2007, the Da Vinci robotic platform, was first used for robot-assisted transaxillary surgery (RATS) by Chung et al. (10). Since then, bilateral axillo-breast and RA approaches have also been developed for the Da Vinci system (10).

In this systematic review, the MIVAT, TOETVA, transaxillary, bilateral axillo-breast and retro-auricular techniques are compared regarding operating time, hospital stay and complications.

## Methods

### Search Strategy

This systematic review was reported according to Preferred Reporting Items for Systematic Reviews and Meta-Analysis guidelines (PRISMA). Eligible studies were identified using Medline, Embase and Web of Science on 11 November 2020. There was no limitation to language or publication date during the search. Combinations of the following keywords and its synonyms were used in the search: “robotic thyroidectomy”, “endoscopic thyroidectomy”, “thyroid surgery”, “RATS”, “TOETVA”, “BABA”, “MIVAT”, “facelift” and “outcome”. In addition, reference lists of included studies were carefully analyzed for related studies. The full search can be found in the **Supplementary Material**.

### Study Selection

Studies were included that reported total operating time, length of hospital stay, outcomes including transient/permanent recurrent laryngeal nerve (RLN) palsy and/or transient/permanent hypocalcemia; and were either a (non-)randomized controlled trial, controlled clinical trial or observational study. Studies with and without control groups were included. Only studies published as original articles and written in English were included. Exclusion criteria were preclinical studies, (systematic) reviews, guidelines, case reports, publications in abstract form only, editorials, letters or conference proceedings.

The titles and abstracts were reviewed by one author (DA) and discussed with another author (LHdV). This was followed by full-text review of potentially eligible studies. Disagreements were discussed and solved with help of a third reviewer (LL) when necessary.

### Quality Analysis of the Studies

Non-randomized and observational studies were assessed using the MINORS checklist, while randomized controlled studies were assessed using the Cochrane risk of bias tool (11, 12).

### Data Extraction

For each study included in the final analysis, the following data was extracted: title, study design, author, year of publication, geographic location, number of patients, patients’ age, gender, surgical approach, type of procedure, total operating time, hospital stay, transient RLN palsy (defined as hoarseness <6 months), permanent RLN palsy (hoarseness >6 months), transient hypocalcemia (defined as hypoparathyroidism <6 months) and permanent hypocalcemia (hypoparathyroidism >6 months). ﻿In most studies, hypoparathyroidism was defined as a reduction in serum iPTH and/or iCa concentration below the normal limit, with or without hypocalcemic symptoms. Due to heterogeneous reporting on cosmetic results, cosmetic satisfaction could not be analyzed in this study.

Both the mean and median were extracted from the articles, if available. Some articles reported results on partial, subtotal or total thyroidectomy; some studies reported results combining multiple extents of surgeries. Means were noted with SD and medians with ranges, if provided in the original article. To provide an impression of operating time and length of hospital stay, the median and interquartile range (IQR) was calculated per technique. This was done using the overall mean per article. The same was done for the incidence of RLN injury and hypocalcemia, using the percentage per article. Articles that described the mean operating time for both partial and total thyroidectomy were included to assess median overall operating time.

### Statistical Analysis

Only studies comparing a minimal invasive technique to conventional thyroidectomy (standard of care) were included in the meta-analysis, studies without control arms were therefore not included. Data was analyzed using Review Manager software, version 5.0 (Cochrane Collaboration, Oxford, UK). Standardized mean difference (SMD) was calculated for continues variables and odds ratio (OR) for the dichotomous variables, including 95% confidence intervals (CI). The Mantel-Haenszel method for pooling was used in case of multiple odds ratios close to one and even sample sizes in both arms. In plots with zero events in one arm without one of these characteristics Peto’s method was used (13). I^2^ above 50% was considered substantial heterogeneity. A random effects model was used for all analyses. *P <*0.05 was considered to be statistically significant.

## Results

### Study Characteristics

**Figure 2:**
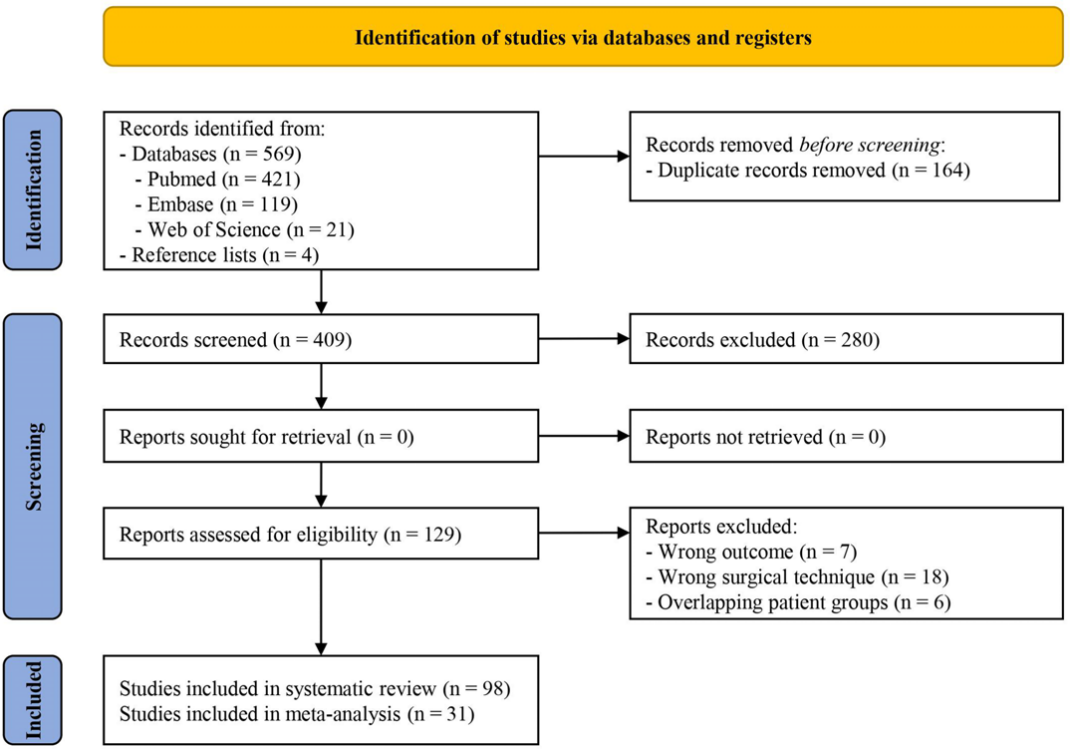
Flowchart of study selection using PRISMA 2020 flow diagram.

A flowchart of study selection is shown in **Figure 2**. A total of 569 articles were retrieved from the databases. Four additional references were found through the reference lists. After duplication removal 409 articles were left. All 409 articles were screened, and 280 articles were excluded based on the abstract, leaving 129 eligible articles. After reading the full text, 31 additional articles were excluded. Ninety-eight articles were included in the qualitative analysis (2, 14–110). Among the 98 articles, there were five randomized studies, one non-randomized study and 92 observational studies. Twenty-one studies were conducted in North America, three in South America, 11 in Europa, one in Africa and 62 in Asia. Study characteristics are shown in **Supplementary Table 1**.

A total of 25,373 participants were included. Median age was 44 years. The vast majority of patients were female. Sample sizes ranged from five to 5,000 participants.

Thirteen articles described the bilateral axillo-breast approach endoscopic thyroidectomy (BABA-ET) in a total number of 1,567 patients, whilst the bilateral axillo-breast approach robotic thyroidectomy (BABA-RT) was described in 15 articles and included 1,498 patients. Eighteen articles described the results of the MIVAT in 4,180 patients. Fourteen articles described the TOETVA in 802 patients. There were five articles that conducted thyroidectomy through the retro-auricular endoscopic approach (RA-ET) in 89 patients. The retro-auricular robotic approach (RA-RT) included 55 patients in two articles. The GTET was described in ten papers and involved 2,017 patients. Thirty articles described the RATS and included the most patients, 15,165 in total.

### Quality of Included Studies

All included studies were considered high quality. Full quality assessment can be found in **Supplementary Tables 2**, **3**.

### Operating Time

The mean or median operating times per study are shown in **Supplementary Table 4**. The median operating times including IQR per technique are shown in **Table 1A**. The median operating time of conventional thyroidectomy was 105.5 minutes. For all minimally invasive techniques, the median operating time was longer than the standard of care, with the exception of the MIVAT, which required 74.3 minutes.

**Table 1:** Operating time and length of hospital stay.

A.	Median	Minimum	Q1	Q3	Maximum	IQR	No. of patients
**Operating time in minutes**							
BABA-ET (16, 33, 34, 36, 65, 66, 69, 80, 85, 103)	154.2	109.3	128.9	165.3	238.2	36.4	1,416
BABA-RT (22, 29, 31, 32, 51, 63, 66, 68, 69, 79, 90)	190.5	118.8	165.1	206.0	290.6	40.9	1,169
MIVAT (26, 39, 46, 47, 62, 73, 86, 87, 98, 99, 110)	74.3	41.0	66.4	91.3	222.5	24.9	3,463
TOETVA (14, 17, 19, 23, 50, 61, 93, 96, 102, 104, 107)	152.0	97.0	121.0	196.1	216.7	75.1	590
RA-ET (17, 25, 27, 35, 92)	143.0	123.4	127.2	148.5	152.0	21.3	89
RA-RT (17, 101)	179.1	167.1	NA	NA	191.0	NA	55
GTET (2, 28, 40, 41, 55, 64, 73, 76, 81)	166.8	129.8	140.6	189.1	297.5	48.6	882
RATS (15, 20, 24, 28, 37, 45, 48, 49, 56, 57, 59, 60, 67, 72, 75, 76, 78, 86, 89, 94, 95)	141.5	101.1	123.2	178.0	232.0	54.8	13,357
Standard of care (2, 14–16, 20, 22, 31, 32, 36, 45, 48, 51, 52, 55, 61, 64, 69, 72, 89, 93, 105, 110)	105.5	70.8	86.1	123.5	156.2	37.4	2,150
**B.**	**Median**	**Minimum**	**Q1**	**Q3**	**Maximum**	**IQR**	**No. of patients**
**Length of hospital stay in days**							
BABA-ET (16, 34, 36, 65, 66, 69, 80, 85, 103, 109)	3.1	2.5	3.0	4.3	6.9	1.3	1,353
BABA-RT (22, 29–32, 51, 63, 66, 68, 69, 74, 109)	3.6	2.9	3.2	4.0	5.1	0.8	1,256
MIVAT (38, 39, 42, 62, 73, 86, 87, 99, 105, 110)	1.9	0.0	1.4	2.7	4.7	1.3	2,937
TOETVA (14, 18, 19, 43, 50, 61, 91, 93, 104, 106, 107)	3.6	0.8	2.3	4.3	5.4	2.0	740
RA-ET (25, 27, 35, 92)	4.5	2.5	3.2	5.6	5.9	2.4	84
RA-RT (101)	NA	NA	NA	NA	NA	NA	0
GTET (2, 28, 55, 73, 76, 79, 84)	3.4	2.2	2.6	5.0	6.5	2.4	1,167
RATS (15, 20, 24, 28, 48, 49, 56, 59, 60, 67, 70, 72, 75, 76, 78, 81, 83, 84, 86, 89, 94, 95)	2.9	0.0	1.2	3.3	5.1	2.1	14,232
Standard of care (2, 14–16, 20, 21, 30, 32, 48, 51, 55, 58, 64, 66, 72, 79, 89, 93, 105, 106, 110)	3.1	0.0	1.0	3.6	5.5	2.6	2,250

Q1, first quartile; Q3, third quartile; IQR, interquartile range; NA, not applicable.

**Figure 3:**
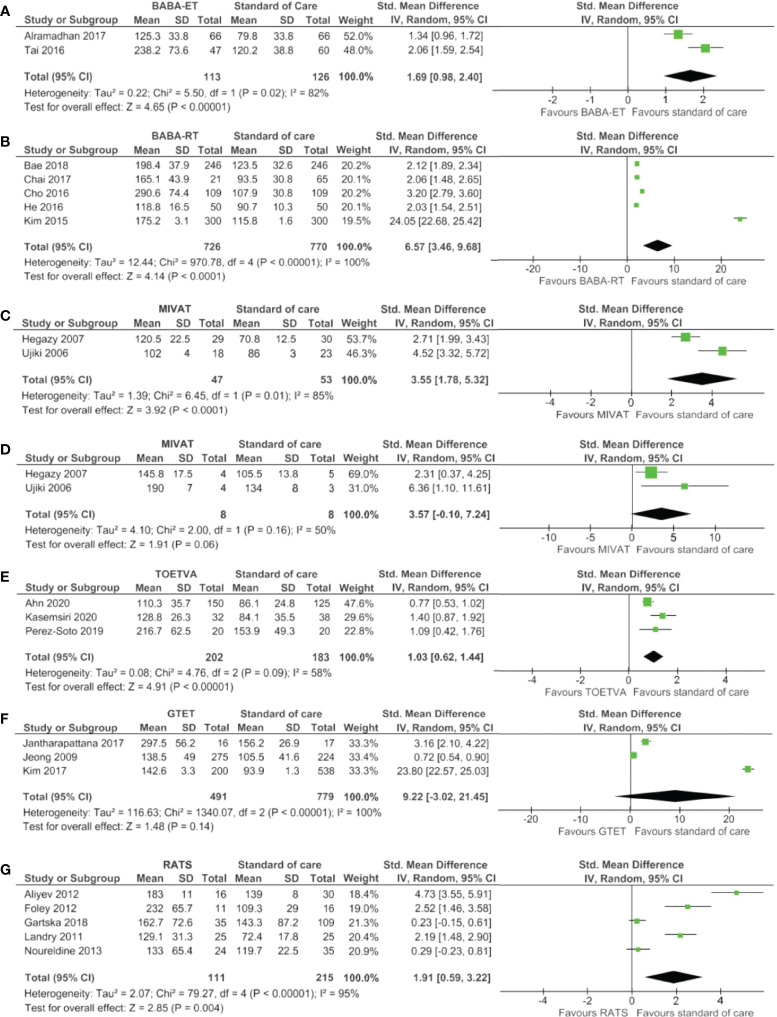
Forest plots of operating time for **(A)** BABA-ET, **(B)** BABA-RT, **(C)** MIVAT lobectomy, **(D)** MIVAT total thyroidectomy, **(E)** TOETVA, **(F)** GTET and **(G)** RATS.

The meta-analysis displayed in **Figure 3** showed a significantly longer operating time for the BABA-ET, BABA-RT, MIVAT (lobectomy), TOETVA, RATS techniques in comparison to standard of care. Operating times for the MIVAT (total thyroidectomy) and GTET were not significantly different compared to standard of care.

### Hospital Stay

Mean or median length of hospital stay per study is shown in **Supplementary Table 4**. The median length of hospital stay including IQR per technique is shown in **Table 1B**. A median hospital stay of approximately three days was reported for all techniques including the standard of care, except for the RA-ET and MIVAT which had a median hospital stay of 4.5 and 1.9 days, respectively. Length of hospital stay was not reported for the RA-RT.

**Figure 4:**
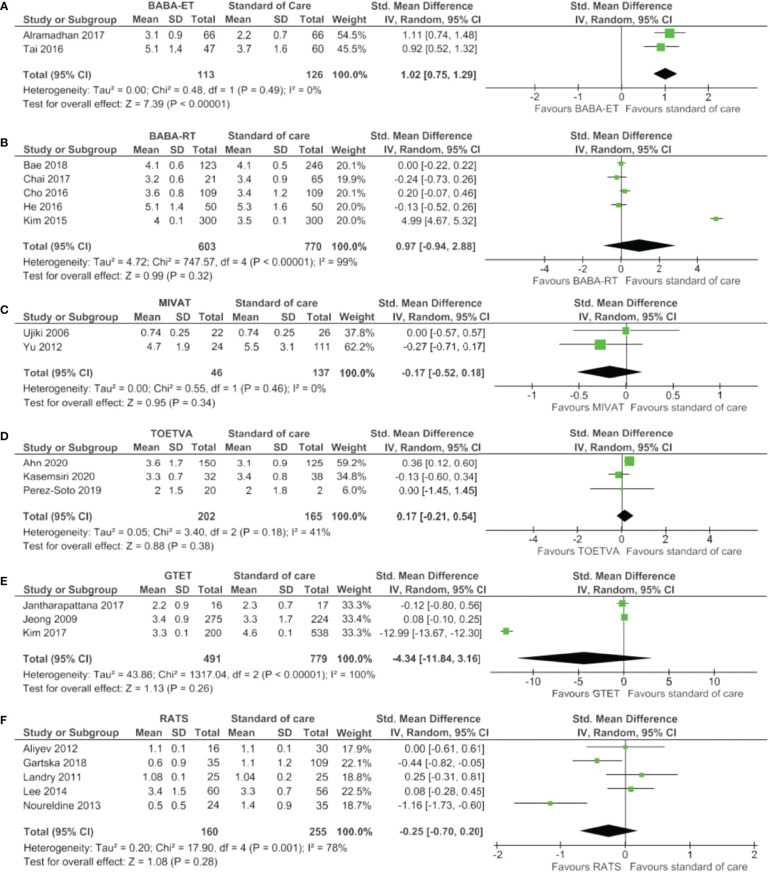
Forests plots of hospital stay for **(A)** BABA-ET, **(B)** BABA-RT, **(C)** MIVAT, **(D)** TOETVA, **(E)** GTET and **(F)** RATS.

The meta-analysis presented in **Figure 4** showed a significantly longer length of hospital stay for the BABA-ET compared to the standard of care. For all other minimally invasive techniques, no significant difference was seen compared to standard of care.

### Transient RLN Injury

Results on transient RLN injury can be found in **Supplementary Table 4** per study and in **Table 2A** per technique. The median incidence of transient RLN injury after BABA-ET (3.7% of 1,491 patients), BABA-RT (3.0% of 1,554 patients), MIVAT (2.5% of 1,727 patients), TOETVA (4.0% of 736 patients) and RATS (3.3% of 15,780) was comparable to the standard of care, which was 3.3% of 2,908 patients. The median incidence of RLN injury was higher in RA-RT and GTET patients (5.0% of 20 patients and 5.3% of 1,921 patients, respectively) and lower after RA-ET (1.1% of 84 patients).

**Table 2:** Incidence of recurrent laryngeal nerve injury.

A.	Median	Minimum	Q1	Q3	Maximum	IQR	No. of cases	No. of patients
**Transient recurrent laryngeal nerve injury in %**								
BABA-ET (16, 33, 34, 36, 54, 65, 66, 69, 80, 85, 103, 109)	3.7	0.0	1.4	7.3	28.0	5.9	162	1,491
BABA-RT (21, 22, 29–32, 51, 63, 66, 68, 69, 74, 79, 90, 109)	3.0	0.0	2.0	6.4	19.0	4.4	77	1,554
MIVAT (26, 38, 39, 42, 44, 45, 52, 62, 71, 73, 86, 98, 99, 105, 110)	2.5	0.0	1.7	4.2	17.0	2.5	86	1,727
TOETVA (14, 18, 19, 23, 43, 61, 91, 93, 102, 106, 107)	4.0	0.0	3.1	7.1	12.5	4.0	32	736
RA-ET (25, 27, 35, 92)	1.1	0.0	0	6.6	11.1	6.6	3	84
RA-RT (101)	5.0	NA	NA	NA	NA	NA	1	20
GTET (28, 40, 41, 55, 59, 64, 73, 76, 81, 84)	5.3	0.0	3.1	6.3	15.0	3.2	85	1,921
RATS (20, 24, 28, 37, 45, 48, 49, 56, 57, 59, 60, 67, 72, 75–78, 81–84, 86, 89, 94, 95, 97, 100)	3.3	0.0	1.8	6.0	20.5	4.2	456	15,780
Standard of care (2, 14, 16, 20, 22, 31, 32, 36, 45, 48, 51, 52, 54, 55, 61, 64, 68, 72, 77, 81, 82, 89, 90, 93, 103, 105, 110)	3.3	0.0	0.0	8.8	19.5	8.8	172	2,908
**B.**	**Median**	**Minimum**	**Q1**	**Q3**	**Maximum**	**IQR**	**No. of cases**	**No. of patients**
**Permanent recurrent laryngeal nerve injury in %**								
BABA-ET (16, 34, 36, 54, 65, 66, 69, 80, 85, 103, 109)	0.0	0.0	0.0	2.1	4.3	2.1	15	1,385
BABA-RT (21, 22, 29–32, 51, 63, 66, 68, 69, 74, 79, 90, 109)	0.0	0.0	0.0	0.7	0.9	0.7	5	1,554
MIVAT (26, 38, 39, 42, 44, 46, 52, 62, 71, 73, 86–88, 98, 99, 105, 110)	0.0	0.0	0.0	1.5	2.9	1.5	45	4,129
TOETVA (14, 18, 19, 23, 43, 61, 91, 93, 96, 102, 106, 107)	0.0	0.0	0.0	1.1	3.1	1.1	6	756
RA-ET (25, 27, 35, 92)	0.0	0.0	0.0	0.0	0.0	0.0	0	84
RA-RT (101)	0.0	NA	NA	NA	NA	NA	0	20
GTET (28, 40, 41, 55, 59, 64, 73, 76, 81, 84)	0.4	0.0	0.0	1.6	6.3	1.6	11	2,033
RATS (20, 24, 28, 37, 48, 49, 56, 57, 59, 60, 67, 72, 75, 76, 78, 83, 84, 86, 89, 94, 95, 97, 100, 108)	0.1	0.0	0.0	0.5	1.3	0.5	59	15,756
Standard of care (2, 14, 16, 20, 22, 31, 32, 36, 48, 51, 52, 54, 55, 61, 64, 68, 72, 88–90, 93, 103, 105, 108, 110)	0.4	0.0	0.0	0.9	4.0	0.9	26	2,970

Q1, first quartile; Q3, third quartile; IQR, interquartile range; NA, not applicable.

**Figure 5:**
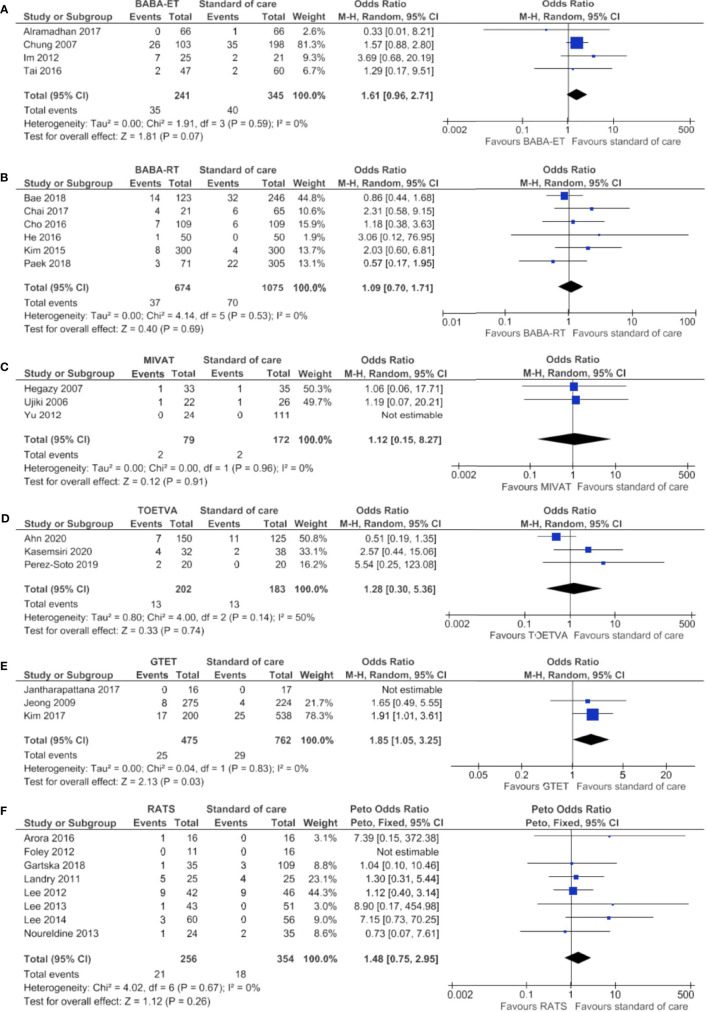
Forests plots of transient recurrent laryngeal nerve injury for **(A)** BABA-ET, **(B)** BABA-RT, **(C)** MIVAT, **(D)** TOETVA, **(E)** GTET and **(F)** RATS.

As shown in **Figure 5**, the meta-analysis showed no significant difference between the minimally invasive techniques and the standard of care considering transient RLN injury.

### Permanent RLN Injury

Permanent RLN injury results are shown in **Supplementary Table 4** per study and in **Table 2B** per technique. The median incidence of permanent RLN injury was 0.4% in 2,970 standard of care patients and was comparable for all minimally invasive techniques, ranging from 0.0% to 0.4%.

**Figure 6:**
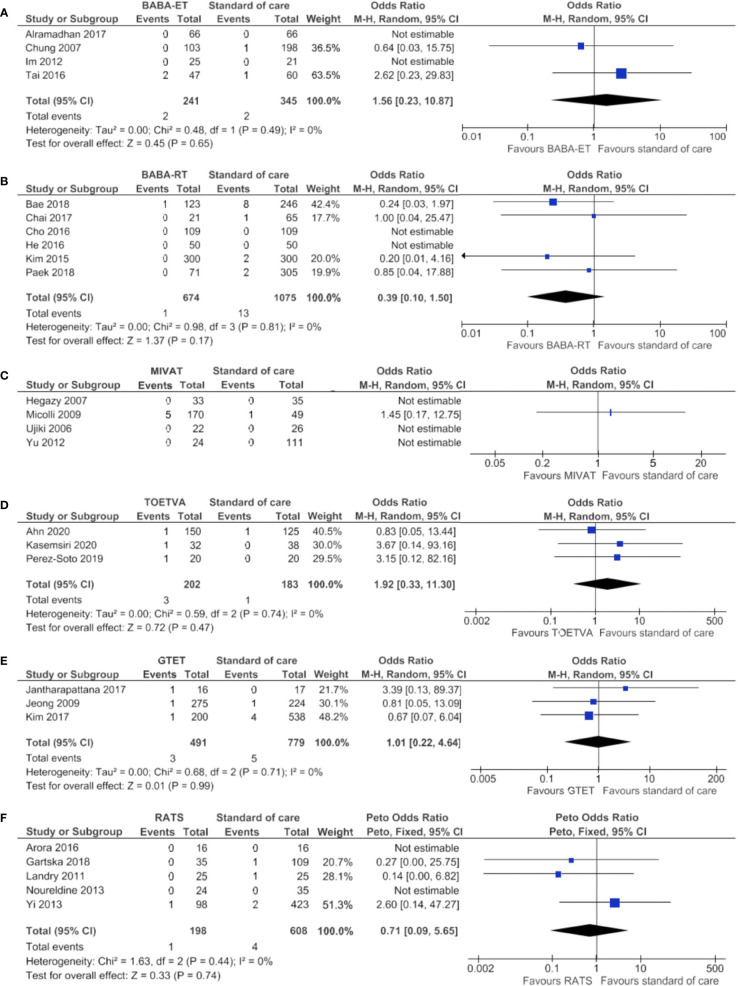
Forests plots of permanent recurrent laryngeal nerve injury for **(A)** BABA-ET, **(B)** BABA-RT, **(C)** MIVAT, **(D)** TOETVA, **(E)** GTET and **(F)** RATS.

**Figure 6** shows the meta-analysis, which confirmed no significant difference for the BABA-ET, BABA-RT, MIVAT, TOETVA, GTET and RATS compared to the standard of care.

### Transient Hypocalcemia

Results on the incidence of transient hypocalcemia can be found in **Supplementary Table 4** per study and in **Table 3A** per technique. The median incidence of transient hypocalcemia seemed to vary between techniques. Transient hypocalcemia occurred in 17.1% of 3,157 patients after standard of care, which was similar to the incidence after RATS (18.4% of 7,002 patients). The median incidence was lower after BABA-ET, MIVAT, TOETVA, RA-ET and GTET (14.0% of 1,208 patients, 5.6% of 3,588 patients, 7.1% of 644 patients, 0.0% of 84 patients and 3.3% of 1,020 patients, respectively) and higher after BABA-RT and RA-RT (21.6% of 1,171 patients and one of three patients, respectively).

**Table 3:** Incidence of hypocalcemia.

A.	Median	Minimum	Q1	Q3	Maximum	IQR	No. of cases	No. of patients
**Transient hypocalcemia in %**								
BABA-ET (16, 33, 34, 36, 53, 54, 65, 66, 69, 80, 85, 103, 109)	14.0	0.0	3.3	28.2	46.2	24.9	259	1,208
BABA-RT (21, 22, 29–32, 51, 63, 66, 68, 69, 74, 79, 90, 109)	21.6	2.8	10.8	31.7	38.6	20.9	283	1,171
MIVAT (26, 38, 39, 42, 44, 46, 47, 52, 62, 71, 73, 87, 98, 99, 110)	5.6	0.0	3.0	10.0	23.4	7.0	231	3,588
TOETVA (14, 18, 19, 43, 91, 93, 102, 106, 107)	7.1	0.0	4.0	21.3	50.0	17.3	64	644
RA-ET (25, 27, 35, 92)	0.0	0.0	0.0	2.8	5.8	2.8	1	84
RA-RT (101)	33.3	NA	NA	NA	NA	NA	1	3
GTET (28, 40, 41, 55, 59, 64, 73, 76, 84)	3.3	0.0	0.0	26.7	33.3	26.7	95	1,020
RATS (15, 24, 28, 37, 45, 48, 49, 56, 57, 59, 60, 67, 70, 75, 76, 78, 81–84, 89, 94, 95, 97, 100, 108)	18.4	0.0	8.3	39.9	51.7	31.7	2,239	7,002
Standard of care (14–16, 22, 31, 32, 36, 45, 48, 51, 52, 54, 55, 64, 68, 81, 82, 89, 90, 93, 103, 107, 110)	17.1	0.0	4.5	34.0	44.6	29.5	631	3,157
**B.**	**Median**	**Minimum**	**Q1**	**Q3**	**Maximum**	**IQR**	**No. of cases**	**No. of patients**
**Permanent hypocalcemia in %**								
BABA-ET (16, 34, 36, 54, 65, 66, 69, 80, 85, 103, 109)	1.3	0.0	0.0	2.1	4.2	2.1	25	1,048
BABA-RT (21, 22, 29–32, 51, 63, 66, 68, 69, 74, 79, 90, 109)	0.9	0.0	0.0	1.7	4.8	1.7	13	1,171
MIVAT (26, 38, 39, 46, 52, 62, 71, 73, 87, 88, 98, 99, 105, 110)	0.0	0.0	0.0	0.0	0.4	0.4	23	3,402
TOETVA (14, 18, 19, 43, 91, 93, 102, 106, 107)	0.0	0.0	0.0	0.7	5.0	0.7	2	644
RA-ET (25, 27, 35, 92)	0.0	0.0	0.0	0.0	0.0	0.0	0	84
RA-RT (101)	0.0	NA	NA	NA	NA	NA	0	3
GTET (28, 40, 41, 55, 59, 64, 73, 76, 84)	0.0	0.0	0.0	0.7	2.7	0.7	4	1,020
RATS (24, 28, 37, 45, 48, 49, 56, 57, 59, 60, 67, 70, 75, 76, 78, 81–84, 89, 94, 95, 97, 100, 108)	0.0	0.0	0.0	0.1	3.1	0.1	45	6,986
Standard of care (14, 16, 22, 31, 32, 36, 45, 48, 51, 52, 54, 55, 64, 68, 88–90, 93, 103, 105, 108, 110)	1.5	0.0	0.0	3.2	6.7	3.2	57	3,024

Q1, first quartile; Q3, third quartile; IQR, interquartile range; NA, not applicable.

**Figure 7:**
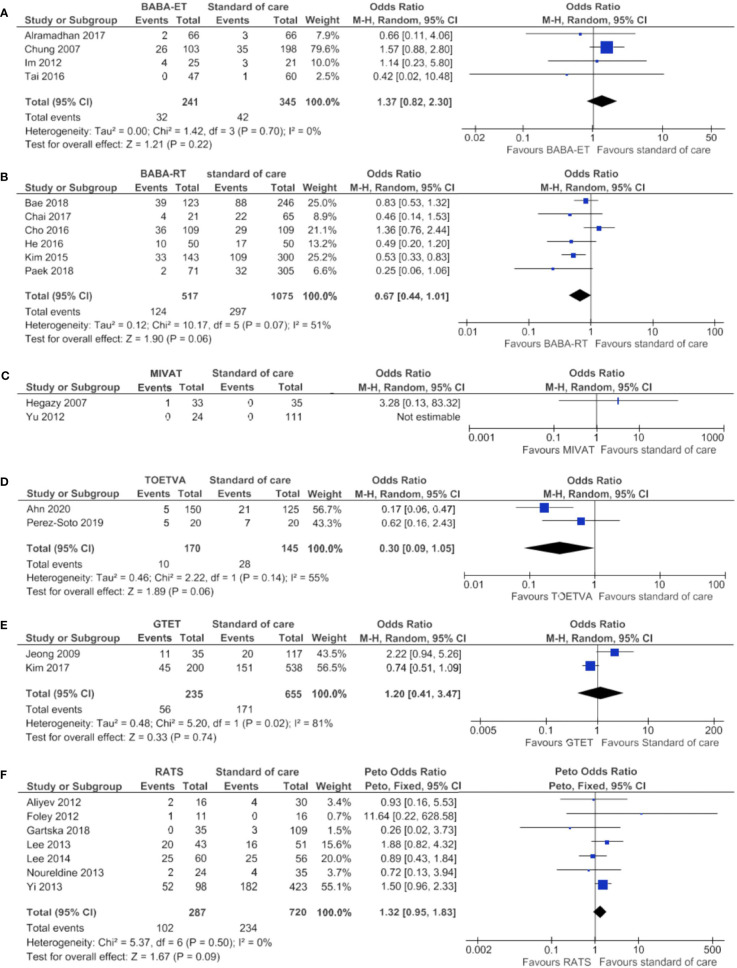
Forests plots of transient hypocalcemia for **(A)** BABA-ET, **(B)** BABA-RT, **(C)** MIVAT, **(D)** TOETVA, **(E)** GTET and **(F)** RATS.

The meta-analysis is displayed in **Figure 7** and showed no significant difference between the minimally invasive techniques and standard of care.

### Permanent Hypocalcemia

Results on permanent hypocalcemia can be found in in **Supplementary Table 4** per study and in **Table 3B** per technique. The median incidence of permanent hypocalcemia after standard of care was 1.5% of 3,024 patients. The median incidence was lower for all other minimally invasive techniques and varied between 0.0% and 1.3%.

**Figure 8:**
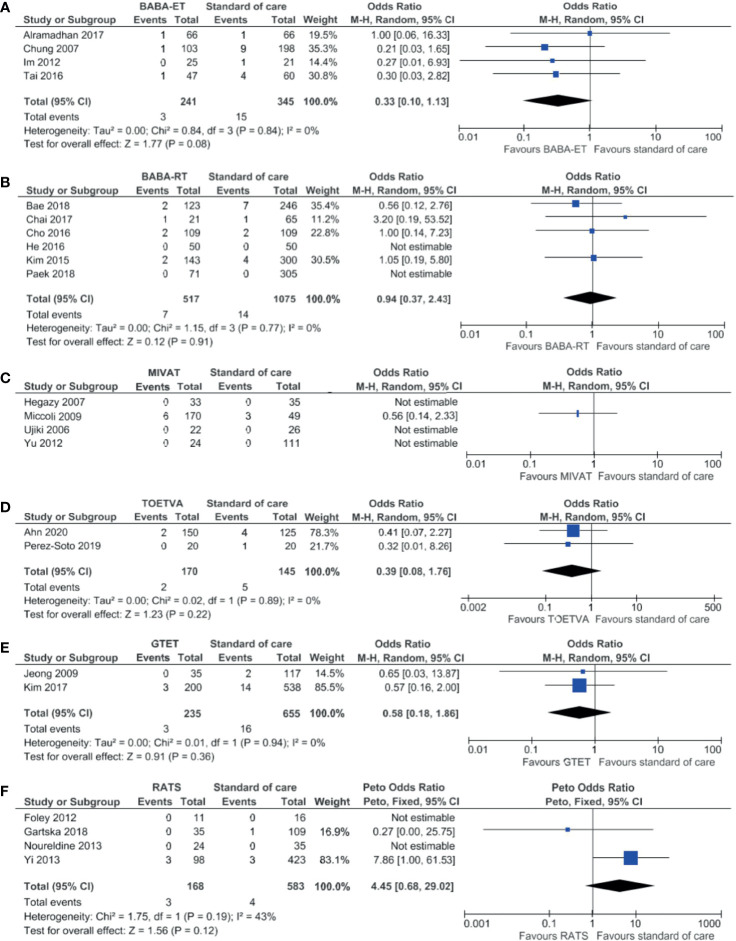
Forests plots of permanent hypocalcemia for **(A)** BABA-ET, **(B)** BABA-RT, **(C)** MIVAT, **(D)** TOETVA, **(E)** GTET and **(F)** RATS.

The meta-analysis in **Figure 8**, showed no significant difference in incidence of transient hypocalcemia after BABA-ET, BABA-RT, MIVAT, TOETVA, GTET and RATS compared to the standard of care.

## Discussion

This systematic review and meta-analysis shows that minimally invasive techniques are not inferior to cervical thyroidectomy with regards to length of hospital stay, transient RLN injury, permanent RLN injury, transient hypocalcemia and permanent hypocalcemia. Prior to this study, multiple systematic reviews have been published regarding either the robotic thyroidectomy techniques or endoscopic thyroidectomy techniques (111–119). However, these systematic reviews combine the different types of techniques. In this systematic review and meta-analysis all minimally invasive techniques were analyzed independently. To the best of our knowledge, this is the largest and most comprehensive systematic review and meta-analysis comparing the eight widely used robotic and endoscopic thyroidectomy techniques.

While the results of our systematic review suggest that the MIVAT is the only minimally invasive technique with a shorter operating time than standard of care, the meta-analysis showed that all techniques, except the GTET have a longer operating time than standard of care. This is to be expected since the docking of the robot platform is time consuming, and in some techniques a subcutaneous route to the thyroid needs to be developed. This longer operating time for the endoscopic and robotic surgical techniques is in concordance with the findings of two other meta-analyses (117, 119).

To provide an overview of the operating time and length of hospital stay, the median overall operating time and hospital stay were calculated. Many studies did not provide the percentage of total thyroidectomies or lobectomies. This makes interpretation of median operating time and length of hospital stay not reliable. The meta-analysis revealed that there was no significant difference regarding the length of hospital stay, between the minimally invasive techniques compared to standard of care, with the exception of shorter hospital stay after BABA-ET. In other studies, also no significant difference was found between the minimally invasive techniques and the conventional approach (112, 114, 116, 117, 119). However, length of hospital stay is not a good metric to assess the best thyroidectomy approach, since it is mostly determined by local prevailing hospital protocols rather than outcome of surgery.

The systematic review showed that the RA-RT and GTET patients had more transient RLN injury compared to the standard of care, while the incidence was lower or comparable after the other techniques. The meta-analysis showed that for the BABA-ET, BABA-RT, RATS, MIVAT and TOETVA no significant difference was found in transient RLN injury when compared to the cervical thyroidectomy. Results from the systematic review regarding the permanent RLN injury showed that the BABA-ET and MIVAT were the only two techniques with more permanent RLN injuries than the standard of care. The meta-analysis, however, showed that none of the minimally invasive technique had a statistically significant difference with the standard of care. Other meta-analyses also found no significant difference in RLN injury (112, 114, 119).

Risk of hypocalcemia is largely influenced by the surgical extent. However, in most articles it was not clearly stated whether hypocalcemia was reported for all surgical extents or solely for total thyroidectomy. Results from this systematic review regarding transient hypocalcemia differed between the techniques. It seemed that the BABA-ET, MIVAT, TOETVA, RA-ET and GTET led to fewer transient hypocalcemia cases than the standard of care. The meta-analysis showed no statistically significant difference between standard of care and minimally invasive techniques. The systematic review showed that all minimally invasive techniques had less cases of permanent hypocalcemia than the standard of care. The meta-analysis showed no significant difference between the minimally invasive techniques and standard of care considering permanent hypocalcemia. Other meta-analyses also found no statistical difference in hypocalcemia between minimally invasive techniques and conventional thyroidectomy (112, 117, 119). Only Jiang et al. reported a lower incidence of hypocalcemia after the endoscopic technique (116). The RA-ET and RA-RT could not be included in the meta-analysis, because of lacking control arms. Therefore the results of RA-ET and RA-RT are solely based on the systematic review.

Minimally invasive techniques can also be used to perform completion thyroidectomies in patients who have undergone minimally invasive thyroid surgery before. Of the studies included in this article, completion thyroidectomy was described by eight studies, using all mentioned techniques except the RA-ET and RA-RT (31, 34, 41, 43, 46, 57, 65, 89). Moreover, several other studies described the safety and feasibility of minimally invasive techniques for the completion thyroidectomy, showing the vast range of application of these techniques (65, 120–122). It is advised to either perform re-operation within two weeks after initial surgery or after two to three months to minimize complications due to adhesions (57, 65, 120).

This systematic review has few limitations. First, there were only a few randomized studies included. The majority of the articles are retrospective in nature, which may have led to selection and reporting bias. Furthermore, only the studies with control groups, a minority of the articles, could be included in the meta-analysis. Lastly, the incidence of permanent RLN injury and permanent hypocalcemia was low in all groups, which made it difficult to show differences between standard of care and minimally invasive techniques.

Previous meta-analyses included mainly studies from Asia (112, 115, 116). Strength of our paper is the inclusion of studies from North America and European countries, in addition to the previously reported Asian articles. Therefore, our results are also applicable to Western populations.

## Conclusion

This study provides a comprehensive review on the most used minimally invasive thyroid surgical techniques. From the systematic review and meta-analysis, it can be concluded that for selected patients, these modern techniques are not inferior to standard of care and are a safe alternative, with the advantage of avoiding a potentially disfiguring scar in the neck.

## Data Availability Statement

The original contributions presented in the study are included in the article/**Supplementary Material**. Further inquiries can be directed to the corresponding author.

## Author Contributions

All authors listed have made a substantial, direct, and intellectual contribution to the work and approved it for publication.

## Conflict of Interest

The authors declare that the research was conducted in the absence of any commercial or financial relationships that could be construed as a potential conflict of interest.

## Publisher’s Note

All claims expressed in this article are solely those of the authors and do not necessarily represent those of their affiliated organizations, or those of the publisher, the editors and the reviewers. Any product that may be evaluated in this article, or claim that may be made by its manufacturer, is not guaranteed or endorsed by the publisher.
