# Supplementary material for: Outcomes of Minimally Invasive Thyroid Surgery – A Systematic Review and Meta-Analysis
Source: Front Endocrinol (Lausanne). 2021 Aug 12;12:719397. doi: 10.3389/fendo.2021.719397 (PMC8387875; doi:10.3389/fendo.2021.719397)
Supplement: Supplementary file 1 [file DataSheet_1.docx]

**Full search**

**PubMed**

((((((((("transoral endoscopic thyroidectomy vestibular approach"[Title/Abstract] OR "TOETVA"[Title/Abstract]) OR (((MIVAT[Title/Abstract]) OR (minimally invasive video-assisted thyroidectomy[Title/Abstract])) OR (minimally invasive video assisted thyroidectomy[Title/Abstract]))) OR ("bilateral axillary breast approach"[Title/Abstract] OR "bilateral axillo-breast approach"[Title/Abstract] OR "bilateral axillo-breast approach"[Title/Abstract] OR "BABA"[Title/Abstract])) OR ("transaxillary"[Title/Abstract] OR "trans-axillary"[Title/Abstract] OR "RATS"[Title/Abstract] OR "RATT"[Title/Abstract] OR "trans axillary"[Title/Abstract])) OR ("facelift"[Title/Abstract] OR "retroauricular"[Title/Abstract] OR "retro-auricular"[Title/Abstract] OR "postauricular"[Title/Abstract] OR "post-auricular"[Title/Abstract] OR "retro auricular"[Title/Abstract] OR "post auricular"[Title/Abstract])) OR ((((((((((((Robot[Title/Abstract]) OR (robotic[Title/Abstract])) OR (robot surgery[Title/Abstract])) OR (robotic surgery[Title/Abstract])) OR (robot assisted[Title/Abstract])) OR (robot-assisted[Title/Abstract])) OR (robotic assisted[Title/Abstract])) OR (robotic-assisted[Title/Abstract])) OR (robot assistance[Title/Abstract])) OR (robot-assistance[Title/Abstract])) OR (robotic-assistance[Title/Abstract])) OR (robotic assistance[Title/Abstract]))) OR (((endoscopic[Title/Abstract]) OR (endoscopic surgery[Title/Abstract])) OR (endoscopic-surgery[Title/Abstract]))) AND (((complication*) OR (safe*)) OR (outcome*))) AND (thyroid neoplasm*[MeSH Terms])) AND ((thyroidectomy[Title/Abstract]) OR (thyroid surgery[Title/Abstract]))

**Embase**

((((((((transoral AND endoscopic AND ('thyroidectomy'/exp OR thyroidectomy) AND vestibular AND approach OR toetva OR mivat OR minimally) AND invasive AND ('video'/exp OR video) AND assisted AND ('thyroidectomy'/exp OR thyroidectomy) OR minimally) AND invasive AND ('video'/exp OR video) AND assisted AND ('thyroidectomy'/exp OR thyroidectomy) OR bilateral) AND axillary AND ('breast'/exp OR breast) AND approach OR bilateral) AND 'axillo breast' AND approach OR bilateral) AND 'axillo breast' AND approach OR baba OR transaxillary OR 'rats'/exp OR rats OR ratt OR 'trans axillary' OR facelift OR retroauricular OR postauricular OR 'retro auricular' OR 'post auricular' OR robotic OR 'robot'/exp OR robot) AND ('surgery'/exp OR surgery) OR robotic) AND ('surgery'/exp OR surgery) OR 'robot assisted' OR 'robotic assisted' OR 'robot assistance' OR 'robotic assistance' OR endoscopic OR 'endoscopic surgery'/exp OR 'endoscopic surgery') AND (complication* OR safe* OR outcome*) AND ('thyroid'/exp OR thyroid) AND neoplasm* AND (('thyroidectomy'/exp OR thyroidectomy OR 'thyroid'/exp OR thyroid) AND ('surgery'/exp OR surgery) OR 'surgery'/exp OR surgery)
