# Supplementary material for: Outcomes of Minimally Invasive Thyroid Surgery – A Systematic Review and Meta-Analysis
Source: Front Endocrinol (Lausanne). 2021 Aug 12;12:719397. doi: 10.3389/fendo.2021.719397 (PMC8387875; doi:10.3389/fendo.2021.719397)
Supplement: Supplementary file 2 [file Table_1.docx]

| **Author and year** | **Country** | **Study design** | **No. of patients** | **Age in years** | **Female %** | **Type of approach** | **Extent of surgery** | **Neck dissection** | **Diagnosis** | | **Reported outcome** |
| --- | --- | --- | --- | --- | --- | --- | --- | --- | --- | --- | --- |
|  |  |  |  |  |  |  |  |  | **Malignant** | **Benign** |  |
| Ahn et al. 2020 (12) | South Korea | Pro | 150 | ﻿43.1 ± 10.9 | 96.7 | TOETVA | Lob, TT | Yes | 150 | 0 | 1, 2, 3 |
| Aliyev et al. 2012 (13) | USA | Pro | 16 | 48 ± 6.0 | 100 | RATS | Lob, TT | No | 11 | 5 | 1, 2, 3 |
| Alramadhan et al. 2017 (14) | South Korea | Retro | 66 | ﻿36.5 ± 9.8 | 97.9 | BABA-ET | Lob, TT | No | 36 | 59 | 1, 2, 3 |
| Alshehri et al. 2017 (15) | USA | Pro | 35  5 | 44 ± 13 | 95.0 | RA-RT  RA-ET | Lob, TT | No | 9 | 31 | 1, 2, 3 |
| Anuwong 2016 (16) | Thailand | NR | 60 | 41.4 ± 12.2 | 95.0 | TOETVA | Lob, TT | No | 2 | 58 | 1, 2, 3 |
| Anuwong et al. 2018 (17) | Thailand | NR | 200 | 40.8 ± 12.1 | 96.0 | TOETVA | Lob, TT | No | 11 | 189 | 1, 2, 3 |
| Arora et al. 2016 (18) | UK | Pro | 16 | 42 ± 10.8 | 93.4 | RATS | Lob | No | 3 | 13 | 1, 2, 3 |
| Bae et al. 2016 (19) | South Korea | Retro | 118 | 39.3 ± 8.7 | 93.2 | BABA-RT | Lob, TT | Yes | 108 | 10 | 1, 2, 3 |
| Bae et al. 2018 (20) | South Korea | Retro | 123 | 40.8 ± 9.0 | 94.3 | BABA-RT | TT | Yes | 123 | 0 | 1, 3 |
| Bakkar et al. 2017 (21) | Jordan | Retro | 5 | 36 (30-45) | 100 | TOETVA | Lob | No | 0 | 5 | 1, 2, 3 |
| Ban et al. 2013 (22) | South Korea | Retro | 3,000 | 39.0 ± 9.1 | 89.7 | RATS | STT, TT | Yes | 3,000 | 0 | 1, 2, 3 |
| Ban et al. 2016 (23) | South Korea | Retro | 8 | 41.1 ± 7.0 | 100 | RA-ET | Lob | Yes | 8 | 0 | 1, 2, 3 |
| Bellotti et al. 2019 (24) | Italy | Retro | 110 | 41.23 | 96.4 | MIVAT | TT | Yes | 10 | 100 | 1, 3 |
| Byeon et al. 2016 (25) | South Korea | Retro | 18 | 42.2 ± 11.4 | 83.3 | RA-ET | Lob, TT | Yes | 12 | 6 | 1, 2, 3 |
| Cabot et al. 2012 (26) | USA | Retro | 30  30 | ﻿42.6 ± 10.0  38.4 ± 9.7 | 96.7  90.0 | GTET  RATS | Lob, TT | Yes | 30  30 | 0  0 | 1, 2 |
| Chae et al. 2020 (27) | South Korea | Retro | 56 | ﻿40.0 ± 9.4 | 89.3 | BABA-RT | Lob | Yes | 56 | 0 | 1, 2, 3 |
| Chai et al. 2017 (28) | South Korea | Retro | 50 | 41.2 ± 9.4 | 92.0 | BABA-RT | Lob, STT, TT | Yes | 37 | 13 | 1, 2, 3 |
| Chai et al. 2017 (29) | South Korea | Retro | 21 | 30.8 ± 12.0 | 90.5 | BABA-RT | Lob, STT | Yes | 21 | 0 | 1, 2, 3 |
| Cho et al. 2016 (30) | South Korea | Retro | 109 | 41.8 ± 9.4 | 86.2 | BABA-RT | Lob, STT, TT | Yes | 126 | 0 | 1, 2, 3 |
| Choe et al. 2007 (31) | South Korea | NR | 110 | NR | NR | BABA-ET | Lob, STT, TT | No | NR | NR | 1, 2, 3 |
| Choi et al. 2012 (32) | South Korea | NR | 512 | ﻿37.8 ± 8.5 | 84.3 | BABA-ET | Lob, STT, TT, CT | Yes | 397 | 106 | 1, 2, 3 |
| Chung et al 2015 (33) | South Korea | Pro | 47 | NR | NR | RA-ET | Lob, TT | NR | NR | NR | 1, 2, 3 |
| Chung et al. 2007 (34) | South Korea | NR | 103 | 38.2 ± 8.2 | 99.0 | BABA-ET | Lob, STT, TT | No | 103 | 0 | 1, 2, 3 |
| Ciabatti et al. 2012 (35) | Italy | NR | 29 | 45 (19-69) | 83.8 | RATS | TT | No | 29 | 0 | 1, 3 |
| Dedivitis et al. 2005 (36) | Brazil | Retro | 12 | 34 (25-68) | 100 | MIVAT | Lob, TT | No | NR | NR | 1, 3 |
| Dobrinja et al. 2009 (37) | Italy | Retro | 47 | 54 (33-75) | 85.1 | MIVAT | Lob, TT | Yes | 40 | 7 | 1, 2, 3 |
| Duncan et al. 2007 (38) | USA | NR | 32 | 33 (19-71) | 93.8 | GTET | Lob, Ist | No | 2 | 30 | 1, 3 |
| Duncan et al. 2009 (39) | USA | Retro | 53 | 34 (19-73) | 88.7 | GTET | CT | No | 2 | 51 | 1, 3 |
| Fan et al. 2009 (40) | China | NR | 300 | 54.6 (23-71) | 88.0 | MIVAT | Lob, TT | Yes | 18 | 282 | 1, 2, 3 |
| Fernandez-Ranvier et al. 2020 (41) | USA | Pro | 149 | 46.9 ± 2.1 | 92.0 | TOETVA | Lob, TT, CT | Yes | 45 | 107 | 1, 2, 3 |
| Fik et al. 2014 (42) | Czech Republic | Pro | 60 | 40 ± 14 | 90.0 | MIVAT | Lob, TT | NR | 17 | 43 | 1, 3 |
| Foley et al. 2012 (43) | USA | Pro | 11 | 46 ± 11.5 | 100 | RATS | Lob, TT | No | 5 | 6 | 1, 2, 3 |
| Frank et al. 2010 (44) | USA | Retro | 583 | 48 ± 16.3 | 83.2 | MIVAT | Lob, TT, CT | NR | NR | NR | 1, 3 |
| Gagner et al. 2001 (45) | USA | NR | 18 | 43 (17-66) | 88.9 | MIVAT | Lob, Ist, STT | No | 2 | 16 | 1, 2 |
| Gartska et al. 2018 (46) | USA | Retro | 35 | 42.1 ± 12.5 | 97.1 | RATS | Lob, TT | Yes | 35 | 0 | 1, 2, 3 |
| Giulianotti et al. 2012 (47) | USA | Pro | 10 | 44 (27-42) | 80.0 | RATS | STT, TT | Yes | 6 | 4 | 1, 2, 3 |
| Guo et al. 2020 (48) | China | Retro | 40 | ﻿31.8 ± 7.0 | 100 | TOETVA | TT | Yes | 40 | 0 | 1, 2 |
| He et al. 2016 (49) | China | Pro | 50 | 40.9 ± 9.8 | 82.0 | BABA-RT | TT | Yes | 50 | 0 | 1, 2, 3 |
| Hegazy et al. 2007 (50) | Egypt | Pro | 33 | 39.8 ± 13.7 | 90.1 | MIVAT | Lob, TT | No | 1 | 32 | 1, 3 |
| Hur et al. 2011 (51) | South Korea | NR | 50 | ﻿38.2 ± 10.1 | 96.0 | BABA-ET | Lob, TT | Yes | 42 | 8 | 1, 2, 3 |
| Im et al. 2012 (52) | South Korea | NR | 25 | ﻿40.8 ± 7.2 | 100 | BABA-ET | TT | Yes | 25 | 0 | 3 |
| Jantharapattana et al. 2017 (2) | Thailand | Pro | 16 | 36.3 ± 12.4 | 100 | GTET | NR | No | 1 | 15 | 1, 2, 3 |
| Jeong et al. 2009 (53) | South Korea | Retro | 275 | ﻿39.6 ± 8.8 | 97.5 | GTET | Lob, STT, TT | Yes | 275 | 0 | 1, 2, 3 |
| Kandil et al. 2012 (54) | USA | Pro | 10 | 38.5 ± 13.5 | 90.0 | RATS | Lob | No | 4 | 6 | 1, 2, 3 |
| Kandil et al. 2012 (55) | USA | Pro | 100 | 45 (13-76) | 86.0 | RATS | Lob, STT, TT, CT | No | 9 | NR | 1, 2, 3 |
| Kang et al. 2009 (56) | South Korea | Retro | 581 | 36.9 ± 9.9 | 98.3 | GTET | Lob, STT, TT | Yes | 410 | 171 | 1, 2, 3 |
| Kang et al. 2009 (57) | South Korea | NR | 338 | 40 (16-69) | 94.4 | RATS | STT, TT | Yes | 332 | 6 | 1, 2, 3 |
| Kang et al. 2011 (58) | South Korea | Pro | 1,000 | 39.1 ± 9.6 | 93.7 | RATS | STT, TT | Yes | 1,000 | 0 | 1, 2, 3 |
| Kasemsiri et al. 2020 (59) | Thailand | Pro | 32 | 28.3 ± 11.3 | 100 | TOETVA | Lob | No | 1 | 31 | 1, 2, 3 |
| Kim et al. 2013 (60) | USA | Retro | 53 | 52 (20-87) | 81.0 | MIVAT | Lob, TT | No | 43 | 10 | 1, 2, 3 |
| Kim et al. 2018 (61) | South Korea | Pro | 43 | 39.8 ± 10.7 | 95.3 | BABA-RT | Lob, TT | Yes | 38 | 5 | 1, 2, 3 |
| Kim et al. 2017 (62) | South Korea | NR | 200 | 39.5 ± 0.8 | 96.0 | GTET | TT | Yes | 200 | 0 | 1, 2, 3 |
| Kim et al. 2010 (63) | South Korea | Retro | 13 | 28.2 ± 5.4 | 92.3 | BABA-ET | CT | No | 13 | 0 | 1, 3 |
| Kim et al. 2017 (64) | South Korea | Retro | 289  289 | 39.6 ± 7.5  39.7 ± 7.7 | 99.3  99.3 | BABA-ET  BABA-RT | Lob, TT | Yes | 289  289 | 0  0 | 1, 2, 3 |
| Kim et al. 2018 (65) | South Korea | Retro | 5,000 | 38.3 ± 9.3 | 88.9 | RATS | STT, TT | Yes | 4,804 | 196 | 1, 2, 3 |
| Kim et al. 2015 (66) | South Korea | NR | 300 | 39.5 ± 0.5 | 94.3 | BABA-RT | Lob, TT | Yes | 300 | 0 | 1, 2, 3 |
| Kim et al. 2011 (67) | South Korea | Retro | 95  69 | 39.9 ± 9.1  41.3 ± 7.8 | 97.9  91.3 | BABA-ET  BABA-RT | TT | Yes | 164 | 0 | 1, 2, 3 |
| Kuppersmith et al. 2010 (68) | USA | Retro | 31 | 38 (20-62) | 96.8 | RATS | Lob, TT | No | 4 | 27 | 1, 2, 3 |
| Lai et al. 2008 (69) | USA | Pro | 40 | 49 (17-77) | 62.5 | MIVAT | Lob, TT | No | 22 | 29 | 1, 3 |
| Landry et al. 2011 (70) | USA | Pro | 25 | 50 (22-62) | 93.0 | RATS | Lob | No | 0 | 25 | 1, 2, 3 |

**Supplementary Table 1.** Characteristics of included studies

*Retro* retrospective; *Pro* prospective; Age is reported as mean ± SD or median (range); *Lob* lobectomy; *Ist* istmusectomy; *STT* subtotal thyroidectomy; *TT* total thyroidectomy; *CT* completion thyroidectomy; Reported outcome is reported as *1* operating time; *2* length of hospital stay; *3* complications; *NR* not reported

| **Author and year** | **Country** | **Study design** | **No. of patients** | **Age in years** | **Females %** | **Type of approach** | **Extent of surgery** | **Neck dissection** | **Diagnosis** | | **Reported outcome** |
| --- | --- | --- | --- | --- | --- | --- | --- | --- | --- | --- | --- |
|  |  |  |  |  |  |  |  |  | **Malignant** | **Benign** |  |
| Lang et al. 2013 (71) | China | NR | 45  96 | 45 (22-60)  43 (19-60) | 91.1  99.0 | MIVAT  GTET | Lob, TT | Yes | 17 | 124 | 1, 2, 3 |
| Lee et al. 2013 (72) | South Korea | Retro | 100 | 40.9 ± 8.9 | 93.0 | BABA-RT | Lob, TT | Yes | 100 | 0 | 1, 2, 3 |
| Lee et al. 2011 (73) | South Korea | Retro | 2,014 | 39.4 ± 9.2 | 91.9 | RATS | Lob, STT | Yes | 1,959 | 55 | 1, 2, 3 |
| Lee et al. 2011 (74) | South Korea | Retro | 96  163 | 39.9 ± 6.5  38.7 ± 8.2 | 97.9  96.3 | GTET  RATS | STT, TT | Yes | 55  152 | 41  11 | 1, 2, 3 |
| Lee et al. 2012 (75) | South Korea | Pro | 42 | 37.9 ± 6.8 | 95.2 | RATS | Lob, TT | Yes | 36 | 6 | 3 |
| Lee et al. 2011 (76) | South Korea | Retro | 1,043 | 39.6 ± 9.2 | 93.1 | RATS | STT, TT | Yes | 1,043 | 0 | 1, 2, 3 |
| Lee et al. 2010 (77) | South Korea | Pro | 109 | 39.0 ± 10 | 85.3 | BABA-RT | Lob, STT, TT | Yes | 109 | 0 | 1, 2, 3 |
| Lee et al. 2013 (78) | South Korea | Retro | 84  45 | 39.4 ± 10.3  40.9 ± 11.2 | 96.4  97.8 | GTET  BABA-ET | Lob | No | 73  42 | 11  3 | 1, 2, 3 |
| Lee et al. 2014 (79) | South Korea | Pro | 60 | 40.5 ± 8.7 | NR | RATS | TT | Yes | 60 | 0 | 2, 3 |
| Lee et al. 2013 (80) | South Korea | Pro | 43 | 39.8 ± 10.2 | NR | RATS | TT | Yes | 43 | 0 | 3 |
| Lee et al. 2011 (81) | South Korea | Retro | 580  570 | 40.3 ± 9.5  38.5 ± 9.2 | 93.2  97.0 | RATS  GTET | STT, TT | Yes | 580 | 0 | 1, 2, 3 |
| Lee et al. 2013 (82) | South Korea | Pro | 400 | 40.6 ± 8.7 | 90.0 | RATS | STT, TT | Yes | 390 | 10 | 1, 2, 3 |
| Liu et al. 2015 (83) | China | Pro | 18 | 37.9 ± 14.4 | 100 | BABA-ET | Lob | No | 3 | 15 | 1, 2, 3 |
| Materazzi et al. 2014 (84) | Italy | Pro | 30  32 | 36.9 ± 9.6  32.5 ± 11.25 | 96.7  96.7 | MIVAT  RATS | Lob | No | 2  3 | 28  29 | 1, 2, 3 |
| Miccoli et al. 2015 (85) | Italy | NR | 2,412 | 40.3 ± 12. 3 | 85.5 | MIVAT | Lob, TT | Yes | 995 | 1,417 | 1, 2, 3 |
| Miccoli et al. 2009 (86) | Italy | NR | 171 | 38.3 ± 12. 5 | 85.3 | MIVAT | TT | Yes | 171 | 0 | 3 |
| Noureldine et al. 2013 (87) | USA | Pro | 24 | 45.4 ± 10.1 | 95.2 | RATS | Lob, TT, CT | No | 24 | 0 | 1, 2, 3 |
| Paek et al. 2018 (88) | South Korea | Retro | 71 | 36.4 ± 9.0 | 93.0 | BABA-RT | TT | Yes | 71 | 0 | 3 |
| Park et al. 2019 (89) | South Korea | Retro | 65 | 42.9 (19-66) | 81.5 | TOETVA | Lob, TT | No | 57 | 8 | 1, 2, 3 |
| Park et al. 2015 (90) | South Korea | Retro | 11 | 36,7 ± 8.4 | 100 | RA-ET | Lob | Yes | 11 | 0 | 1, 2, 3 |
| Perez-Soto et al. 2019 (91) | Mexico | Retro | 20 | 48.1 ± 15.7 | 90.0 | TOETVA | Lob, STT, TT | Yes | 18 | 2 | 1, 2, 3 |
| Piccoli et al. 2019 (92) | Italy | NR | 449 | 44.1 ± 12. 1 | 86.1 | RATS | Lob, TT | No | 112 | 335 | 1, 2, 3 |
| Prete et al. 2019 (93) | Italy | Pro | 12 | 44.9 (21-63) | 100 | RATS | Lob, TT | No | 0 | 12 | 1, 2, 3 |
| Razavi et al. 2017 (94) | USA | Retro | 20 | 41.3 ± 12.2 | 80.0 | TOETVA | Lob | No | 5 | 15 | 1, 3 |
| Ryu et al. 2010 (95) | South Korea | NR | 281 | ﻿38.6 ± 8.8 | 92.2 | RATS | Lob, STT, TT | Yes | 281 | 0 | 1, 2, 3 |
| Schabram et al. 2004 (96) | Germany | Pro | 196 | 46 (13-76) | 70.9 | MIVAT | Lob | No | 12 | 184 | 1, 3 |
| Shan et al. 2012 (97) | China | Pro | 24 | 32.1 ± 9.1 | 95.8 | MIVAT | Lob, STT | No | 0 | 24 | 1, 2, 3 |
| Stang et al. 2018 (98) | USA | Pro | 282 | 41.2 ± 12.0 | 98.9 | RATS | Lob, TT | Yes | 133 | 148 | 1, 2, 3 |
| Sung et al. 2016 (99) | South Korea | Retro | 20 | 42.3 ± 10.8 | 75.0 | RA-RT | Lob, TT | Yes | 15 | 5 | 1, 3 |
| Tae et al. 2018 (100) | South Korea | NR | 14 | 33.9 ± 12.5 | 57.1 | TOETVA | Lob, TT | Yes | 10 | 4 | 1, 3 |
| Tai et al. 2016 (101) | China | Retro | 47 | 46.4 ± 10.9 | 91.5 | BABA-ET | Lob, Ist, TT | No | 5 | 42 | 1, 2, 3 |
| Tesseroli et al. 2018 (102) | Brazil | Pro | 9 | 47.4 ± 10.6 | 100 | TOETVA | STT, TT | No | 2 | 7 | 1, 2 |
| Ujiki et al. 2006 (103) | USA | Retro | 22 | 45 ± 3 | 95.5 | MIVAT | Lob, TT | No | 2 | 20 | 1, 2 |
| Wang et al. 2018 (104) | China | NR | 18 | 33 (22-45) | 100 | TOETVA | Lob, Ist, STT, TT | Yes | 12 | 6 | 1, 2, 3 |
| Yi et al. 2017 (105) | South Korea | Retro | 20 | 50.8 ± 11.3 | 95.0 | TOETVA | Lob, Ist, TT | Yes | 20 | 0 | 1, 2, 3 |
| Yi et al. 2013 (106) | South Korea | Retro | 98 | 42.2 ± 8.2 | 100 | RATS | TT | Yes | 98 | 0 | 1, 2, 3 |
| Yoo et al. 2012 (107) | South Korea | Retro | 165  46 | 38.9 ± 9.1  37.4 ± 8.1 | 100  100 | BABA-ET  BABA-RT | Lob, TT | Yes | 148  44 | 17  2 | 1, 2, 3 |
| Yu et al. 2012 (108) | China | Retro | 24 | 45 ± 10 | NR | MIVAT | NR | Yes | 24 | 0 | 1, 3 |

**Supplementary Table 1. *(continued)*** Characteristics of included studies

*Retro* retrospective; *Pro* prospective; Age is reported as mean ± SD or median (range); *Lob* lobectomy; *Ist* istmusectomy; *STT* subtotal thyroidectomy; *TT* total thyroidectomy; *CT* completion thyroidectomy; Reported outcome is reported as *1* operating time; *2* length of hospital stay; *3* complications; *NR* not reported
