# Supplementary material for: Outcomes of Minimally Invasive Thyroid Surgery – A Systematic Review and Meta-Analysis
Source: Front Endocrinol (Lausanne). 2021 Aug 12;12:719397. doi: 10.3389/fendo.2021.719397 (PMC8387875; doi:10.3389/fendo.2021.719397)
Supplement: Supplementary file 3 [file Table_2.docx]

| **Author and year** | **Clearly stated aim** | **Inclusion of consecutive patients** | **Prospective collection of data** | **Appropriate endpoints** | **Unbiased assessment of study endpoint** | **Appropriate follow-up period** | **Loss to follow-up <5%** | **Prospective study size calculation** | **Adequate control group*** | **Contemporary groups*** | **Baseline equivalence of groups*** | **Adequate statistical analyses*** | **Total score** |
| --- | --- | --- | --- | --- | --- | --- | --- | --- | --- | --- | --- | --- | --- |
| Ahn et al. 2020 (12) | 2 | 1 | 2 | 1 | 2 | 2 | 2 | 1 | 2 | 1 | 1 | 1 | 18/24 |
| Aliyev et al. 2012 (13) | 2 | 2 | 2 | 2 | 2 | 2 | 2 | 1 | NA | NA | NA | NA | 15/16 |
| Alramadhan et al. 2017 (14) | 2 | 2 | 1 | 2 | 0 | 2 | 2 | 1 | 2 | 2 | 2 | 1 | 19/24 |
| Alshehri et al. 2017 (15) | 2 | 2 | 2 | 2 | 2 | 2 | 2 | 1 | NA | NA | NA | NA | 15/16 |
| Anuwong 2016 (16) | 2 | 2 | 0 | 2 | 2 | 2 | 2 | 0 | NA | NA | NA | NA | 12/16 |
| Anuwong et al. 2018 (17) | 2 | 2 | 0 | 2 | 2 | 2 | 2 | 0 | NA | NA | NA | NA | 12/16 |
| Arora et al. 2016 (18) | 2 | 2 | 2 | 2 | 2 | 2 | 2 | 0 | NA | NA | NA | NA | 14/16 |
| Bae et al. 2016 (19) | 2 | 2 | 1 | 2 | 2 | 2 | 2 | 1 | NA | NA | NA | NA | 14/16 |
| Bae et al. 2018 (20) | 2 | 2 | 1 | 2 | 1 | 2 | 2 | 1 | 2 | 0 | 2 | 2 | 19/24 |
| Bakkar et al. 2017 (21) | 2 | 2 | 1 | 2 | 2 | 2 | 2 | 0 | NA | NA | NA | NA | 13/16 |
| Ban et al. 2013 (22) | 2 | 2 | 1 | 2 | 2 | 2 | 2 | 1 | NA | NA | NA | NA | 14/16 |
| Ban et al. 2016 (23) | 2 | 2 | 1 | 2 | 1 | 2 | 2 | 1 | NA | NA | NA | NA | 13/16 |
| Bellotti et al. 2019 (24) | 2 | 2 | 1 | 2 | 2 | 2 | 0 | 0 | NA | NA | NA | NA | 11/16 |
| Byeon et al. 2016 (25) | 2 | 2 | 1 | 1 | 1 | 2 | 2 | 1 | NA | NA | NA | NA | 12/16 |
| Cabot et al. 2012 (26) | 2 | 2 | 1 | 1 | 2 | 2 | 2 | 1 | 2 | 2 | 2 | 1 | 20/24 |
| Chae et al. 2020 (27) | 2 | 2 | 1 | 2 | 2 | 2 | 2 | 2 | 0 | 2 | 2 | 1 | 20/24 |
| Chai et al. 2017 (28) | 2 | 2 | 1 | 2 | 2 | 2 | 2 | 1 | 0 | 2 | 2 | 2 | 20/24 |
| Chai et al. 2017 (29) | 2 | 2 | 1 | 2 | 2 | 2 | 2 | 1 | 2 | 2 | 2 | 2 | 22/24 |
| Cho et al. 2016 (30) | 2 | 2 | 1 | 2 | 2 | 2 | 2 | 1 | 2 | 2 | 2 | 2 | 22/24 |
| Choe et al. 2007 (31) | 2 | 2 | 0 | 1 | 2 | 2 | 1 | 0 | 2 | 2 | 2 | 2 | 18/24 |
| Choi et al. 2012 (32) | 2 | 2 | 0 | 2 | 2 | 2 | 2 | 0 | NA | NA | NA | NA | 12/16 |
| Chung et al 2015 (33) | 2 | 1 | 2 | 2 | 2 | 2 | 2 | 1 | 2 | 2 | 2 | 1 | 21/24 |
| Chung et al. 2007 (34) | 2 | 2 | 0 | 2 | 2 | 2 | 1 | 1 | 2 | 2 | 2 | 2 | 20/24 |
| Ciabatti et al. 2012 (35) | 2 | 2 | 0 | 2 | 2 | 2 | 2 | 0 | NA | NA | NA | NA | 12/16 |
| Dedivitis et al. 2005 (36) | 2 | 1 | 1 | 2 | 1 | 2 | 2 | 0 | NA | NA | NA | NA | 11/16 |
| Dobrinja et al. 2009 (37) | 2 | 2 | 1 | 2 | 2 | 2 | 2 | 0 | NA | NA | NA | NA | 13/16 |
| Duncan et al. 2007 (38) | 2 | 2 | 0 | 2 | 2 | 1 | 2 | 0 | NA | NA | NA | NA | 11/16 |
| Duncan et al. 2009 (39) | 2 | 2 | 1 | 1 | 2 | 1 | 0 | 0 | NA | NA | NA | NA | 9/16 |
| Fan et al. 2009 (40) | 2 | 2 | 0 | 1 | 2 | 2 | 2 | 0 | NA | NA | NA | NA | 11/16 |
| Fernandez-Ranvier et al. 2020 (41) | 2 | 2 | 2 | 1 | 2 | 2 | 2 | 1 | NA | NA | NA | NA | 14/16 |
| Fik et al. 2014 (42) | 1 | 2 | 2 | 2 | 2 | 2 | 2 | 1 | 0 | 2 | 2 | 1 | 19/24 |
| Foley et al. 2012 (43) | 2 | 2 | 2 | 2 | 2 | 2 | 2 | 1 | 2 | 2 | 2 | 1 | 22/24 |
| Frank et al. 2010 (44) | 1 | 2 | 1 | 2 | 2 | 2 | 2 | 1 | NA | NA | NA | NA | 13/16 |
| Gagner et al. 2001 (45) | 1 | 2 | 0 | 2 | 2 | 2 | 2 | 1 | 2 | 1 | 1 | 1 | 17/24 |
| Gartska et al. 2018 (46) | 2 | 2 | 2 | 2 | 2 | 2 | 2 | 1 | 2 | 2 | 2 | 1 | 22/24 |
| Giulianotti et al. 2012 (47) | 1 | 2 | 2 | 1 | 2 | 2 | 2 | 0 | NA | NA | NA | NA | 12/16 |
| Guo et al. 2020 (48) | 2 | 2 | 1 | 2 | 2 | 2 | 2 | 1 | 2 | 2 | 2 | 2 | 22/24 |
| Hur et al. 2011 (51) | 2 | 2 | 0 | 2 | 2 | 2 | 2 | 1 | 1 | 2 | 2 | 2 | 20/24 |
| Im et al. 2012 (52) | 2 | 2 | 0 | 2 | 2 | 2 | 2 | 2 | 2 | 2 | 2 | 2 | 22/24 |
| Jeong et al. 2009 (53) | 2 | 2 | 1 | 2 | 2 | 2 | 1 | 1 | 2 | 2 | 2 | 0 | 19/24 |
| Kandil et al. 2012 (54) | 2 | 2 | 2 | 2 | 1 | 2 | 2 | 0 | NA | NA | NA | NA | 13/16 |
| Kandil et al. 2012 (55) | 2 | 2 | 2 | 2 | 2 | 2 | 2 | 1 | NA | NA | NA | NA | 15/16 |
| Kang et al. 2009 (56) | 2 | 2 | 1 | 2 | 2 | 2 | 2 | 0 | NA | NA | NA | NA | 13/16 |
| Kang et al. 2009 (57) | 1 | 2 | 0 | 2 | 2 | 2 | 2 | 0 | NA | NA | NA | NA | 11/16 |
| Kang et al. 2011 (58) | 2 | 2 | 2 | 2 | 2 | 2 | 2 | 0 | NA | NA | NA | NA | 14/16 |
| Kasemsiri et al. 2020 (59) | 2 | 2 | 2 | 2 | 2 | 2 | 2 | 1 | 2 | 2 | 2 | 1 | 22/24 |
| Kim et al. 2013 (60) | 2 | 2 | 1 | 2 | 2 | 2 | 2 | 0 | NA | NA | NA | NA | 13/16 |
| Kim et al. 2018 (61) | 2 | 2 | 2 | 2 | 2 | 2 | 2 | 1 | 0 | 2 | 2 | 2 | 21/24 |

**Supplementary Table 2.** Risk of bias assessment using MINORS criteria

The items are scored *0* not reported; *1* reported but inadequate; *2* reported and adequate. The global ideal score is 16 for non-comparative studies and 24 for comparative studies. * Only applicable for studies with a control group

| **Author and year** | **Clearly stated aim** | **Inclusion of consecutive patients** | **Prospective collection of data** | **Appropriate endpoints** | **Unbiased assessment of study endpoint** | **Appropriate follow-up period** | **Loss to follow-up <5%** | **Prospective study size calculation** | **Adequate control group*** | **Contemporary groups*** | **Baseline equivalence of groups*** | **Adequate statistical analyses*** | **Total score** |
| --- | --- | --- | --- | --- | --- | --- | --- | --- | --- | --- | --- | --- | --- |
| Kim et al. 2017 (62) | 2 | 2 | 0 | 2 | 2 | 2 | 2 | 1 | 2 | 2 | 2 | 1 | 20/24 |
| Kim et al. 2010 (63) | 2 | 2 | 1 | 2 | 2 | 2 | 2 | 0 | NA | NA | NA | NA | 13/16 |
| Kim et al. 2017 (64) | 2 | 2 | 1 | 2 | 2 | 2 | 1 | 1 | 1 | 2 | 2 | 2 | 20/24 |
| Kim et al. 2018 (65) | 2 | 2 | 1 | 2 | 2 | 2 | 2 | 0 | NA | NA | NA | NA | 13/16 |
| Kim et al. 2015 (66) | 2 | 2 | 0 | 2 | 2 | 2 | 2 | 1 | 2 | 2 | 2 | 2 | 21/24 |
| Kim et al. 2011 (67) | 2 | 2 | 1 | 2 | 2 | 2 | 2 | 1 | 2 | 2 | 2 | 2 | 22/24 |
| Kuppersmith et al. 2010 (68) | 2 | 2 | 1 | 2 | 2 | 1 | 2 | 0 | NA | NA | NA | NA | 12/16 |
| Lai et al. 2008 (69) | 2 | 2 | 2 | 2 | 2 | 1 | 2 | 0 | NA | NA | NA | NA | 13/16 |
| Landry et al. 2011 (70) | 2 | 2 | 2 | 2 | 2 | 2 | 2 | 1 | 2 | 2 | 2 | 1 | 22/24 |
| Lang et al. 2013 (71) | 2 | 1 | 0 | 2 | 2 | 2 | 2 | 1 | 1 | 2 | 2 | 1 | 18/24 |
| Lee et al. 2013 (72) | 2 | 2 | 1 | 2 | 2 | 2 | 2 | 0 | NA | NA | NA | NA | 13/16 |
| Lee et al. 2011 (73) | 2 | 2 | 1 | 2 | 2 | 2 | 2 | 1 | 2 | 0 | 0 | 2 | 18/24 |
| Lee et al. 2011 (74) | 2 | 2 | 1 | 2 | 2 | 1 | 2 | 1 | 1 | 2 | 2 | 2 | 20/24 |
| Lee et al. 2012 (75) | 2 | 2 | 2 | 2 | 2 | 2 | 2 | 1 | 2 | 2 | 2 | 2 | 23/24 |
| Lee et al. 2011 (76) | 2 | 2 | 1 | 2 | 2 | 2 | 2 | 0 | NA | NA | NA | NA | 13/16 |
| Lee et al. 2010 (77) | 2 | 2 | 2 | 2 | 2 | 2 | 2 | 0 | NA | NA | NA | NA | 14/16 |
| Lee et al. 2013 (78) | 1 | 2 | 1 | 2 | 2 | 2 | 2 | 1 | 1 | 2 | 2 | 2 | 20/24 |
| Lee et al. 2014 (79) | 2 | 2 | 2 | 2 | 2 | 2 | 2 | 1 | 2 | 2 | 2 | 2 | 23/24 |
| Lee et al. 2013 (80) | 2 | 2 | 2 | 2 | 2 | 2 | 2 | 1 | 2 | 2 | 2 | 2 | 23/24 |
| Lee et al. 2011 (81) | 2 | 2 | 1 | 2 | 2 | 2 | 1 | 1 | 2 | 2 | 2 | 2 | 21/24 |
| Lee et al. 2013 (82) | 2 | 2 | 2 | 2 | 2 | 2 | 2 | 1 | NA | NA | NA | NA | 15/16 |
| Liu et al. 2015 (83) | 2 | 2 | 2 | 2 | 2 | 2 | 2 | 0 | NA | NA | NA | NA | 14/16 |
| Miccoli et al. 2015 (85) | 2 | 2 | 0 | 2 | 2 | 2 | 1 | 0 | NA | NA | NA | NA | 11/16 |
| Miccoli et al. 2009 (86) | 2 | 0 | 0 | 2 | 2 | 2 | 2 | 1 | 2 | 2 | 2 | 2 | 19/24 |
| Noureldine et al. 2013 (87) | 2 | 2 | 2 | 2 | 2 | 2 | 2 | 1 | 2 | 2 | 2 | 2 | 23/24 |
| Paek et al. 2018 (88) | 2 | 2 | 1 | 2 | 2 | 2 | 2 | 1 | NA | NA | NA | NA | 14/16 |
| Park et al. 2019 (89) | 2 | 2 | 1 | 2 | 2 | 2 | 2 | 0 | NA | NA | NA | NA | 13/16 |
| Park et al. 2015 (90) | 2 | 2 | 1 | 2 | 1 | 2 | 2 | 0 | NA | NA | NA | NA | 12/16 |
| Perez-Soto et al. 2019 (91) | 2 | 2 | 1 | 2 | 2 | 2 | 2 | 1 | 2 | 0 | 2 | 2 | 20/24 |
| Piccoli et al. 2019 (92) | 2 | 2 | 0 | 2 | 2 | 2 | 2 | 0 | NA | NA | NA | NA | 12/16 |
| Prete et al. 2019 (93) | 1 | 2 | 2 | 2 | 2 | 2 | 2 | 0 | NA | NA | NA | NA | 13/16 |
| Razavi et al. 2017 (94) | 2 | 2 | 1 | 2 | 1 | 2 | 2 | 1 | 1 | 2 | 2 | 2 | 20/24 |
| Ryu et al. 2010 (95) | 1 | 2 | 0 | 1 | 2 | 2 | 2 | 1 | NA | NA | NA | NA | 11/16 |
| Schabram et al. 2004 (96) | 2 | 2 | 2 | 2 | 2 | 2 | 2 | 0 | NA | NA | NA | NA | 14/16 |
| Stang et al. 2018 (98) | 2 | 2 | 2 | 2 | 2 | 2 | 2 | 1 | NA | NA | NA | NA | 15/16 |
| Sung et al. 2016 (99) | 2 | 2 | 1 | 2 | 2 | 2 | 2 | 1 | 0 | 2 | 2 | 1 | 19/24 |
| Tae et al. 2018 (100) | 2 | 2 | 0 | 2 | 2 | 2 | 2 | 1 | 1 | 2 | 2 | 0 | 18/24 |
| Tai et al. 2016 (101) | 2 | 2 | 1 | 2 | 2 | 2 | 2 | 1 | 2 | 2 | 2 | 2 | 22/24 |
| Tesseroli et al. 2018 (102) | 2 | 2 | 2 | 1 | 1 | 2 | 2 | 0 | NA | NA | NA | NA | 12/16 |
| Ujiki et al. 2006 (103) | 2 | 0 | 1 | 1 | 2 | 2 | 2 | 1 | 2 | 1 | 2 | 2 | 18/24 |
| Wang et al. 2018 (104) | 1 | 2 | 0 | 2 | 1 | 2 | 2 | 0 | NA | NA | NA | NA | 10/16 |
| Yi et al. 2017 (105) | 2 | 2 | 1 | 2 | 2 | 2 | 2 | 0 | NA | NA | NA | NA | 13/16 |
| Yi et al. 2013 (106) | 2 | 2 | 1 | 2 | 2 | 2 | 2 | 1 | 2 | 2 | 2 | 2 | 22/24 |
| Yoo et al. 2012 (107) | 2 | 2 | 1 | 2 | 2 | 2 | 2 | 1 | 1 | 2 | 2 | 2 | 21/24 |
| Yu et al. 2012 (108) | 2 | 2 | 1 | 2 | 2 | 2 | 2 | 1 | 2 | 2 | 2 | 2 | 22/24 |

**Supplementary Table 2. *(continued)*** Risk of bias assessment using MINORS criteria

The items are scored *0* not reported; *1* reported but inadequate; *2* reported and adequate. The global ideal score is 16 for non-comparative studies and 24 for comparative studies. * Only applicable for studies with a control group
