# Supplementary material for: Outcomes of Minimally Invasive Thyroid Surgery – A Systematic Review and Meta-Analysis
Source: Front Endocrinol (Lausanne). 2021 Aug 12;12:719397. doi: 10.3389/fendo.2021.719397 (PMC8387875; doi:10.3389/fendo.2021.719397)
Supplement: Supplementary file 4 [file Table_3.docx]

**Supplementary Table 3.** Risk of bias assessment using Cochrane risk of bias

*+* low risk of bias; *-* high risk of bias; *?* unclear risk of bias; *LRoB* low risk of bias

| **Author and year** | **Random sequence generation** | **Allocation concealment** | **Blinding of participants and personnel** | **Incomplete outcome data** | **Selective reporting** | **Other bias** | **Conclusion** |
| --- | --- | --- | --- | --- | --- | --- | --- |
| He et al. 2016 (49) | + | + | + | + | ? | + | LRoB |
| Hegazy et al. 2007 (50) | + | ? | ? | + | + | + | LRoB |
| Jantharapattana et al. 2017 (2) | + | + | ? | ? | - | + | LRoB |
| Materazzi et al. 2014 (84) | + | ? | ? | + | + | + | LRoB |
| Shan et al. 2012 (97) | + | ? | ? | + | + | + | LRoB |
