# Supplementary material for: Outcomes of Minimally Invasive Thyroid Surgery – A Systematic Review and Meta-Analysis
Source: Front Endocrinol (Lausanne). 2021 Aug 12;12:719397. doi: 10.3389/fendo.2021.719397 (PMC8387875; doi:10.3389/fendo.2021.719397)
Supplement: Supplementary file 5 [file Table_4.docx]

| **Supplementary Table 4.** Surgical and post-surgical outcomes | | | | | | |
| --- | --- | --- | --- | --- | --- | --- |
| **A.** | **Operating time in minutes** | **Hospital stay in days** | **Transient RLN palsy % (no. of patients)** | **Permanent RLN palsy % (no. of patients)** | **Transient hypocalcemia % (no. of patients)** | **Permanent hypocalcemia % (no. of patients)** |
| **BABA-ET** | | | | | | |
| Alramadhan et al. 2017 (14) | 125.3 ± 33.8 | 3.1 ± 0.9 | 0.0 (0/66) | 0.0 (0/66) | 3.0 (2/66) | 1.5 (1/66) |
| Choe et al. 2007 (31) | 165.3 ± 43.5 | NR | 3.6 (4/110) | NR | 3.6 (4/110) | NR |
| Choi et al. 2012 (32) | 152.5 ± 39.9 | 3.3 ± 0.8 | 20.3 (104/512) | 1.7 (9/512) | 31.1 (125/402) | 4.2 (17/402) |
| Chung et al. 2007 (34) | 165.1 | 3.0 | 2.5 (26/103) | 0.0 (0/103) | 25.2 (26/103) | 1.0 (1/103) |
| Hur et al. 2011 (51) | 102.6 ± 25.0 (Lob)  131.0 ± 28.0 (TT) | 4.2 ± 0.8 (Lob)  4.1 ± 1.1 (TT) | NR | NR | 12.0 (6/50) | NR |
| Im et al. 2012 (52) | NR | NR | 28 (7/25) | 0.0 (0/25) | 16.0 (4/25) | 0.0 (0/25) |
| Kim et al. 2010 (63) | 109.3 ± 23.3 | 3.0 ± 0.0 | 0.0 (0/13) | 0.0 (0/13) | 46.2 (6/13) | 0 (0/13) |
| Kim et al. 2017 (64) | 128.9 ± 40.0 | 4.3 ± 1.8 | 3.8 (11/289) | 0.3 (1/289) | 33.3 (44/114) | 1.8 (2/114) |
| Kim et al. 2011 (67) | 136 ± 18.6 | 3.1 ± 0.9 | 2.1 (2/95) | 2.1 (2/95) | 25.3 (24/95) | 3.2 (3/95) |
| Lee et al. 2013 (78) | 184.9 ± 62.7 | 6.9 ± 1.5 | 8.9 (4/45) | 2.2 (1/45) | NR | NR |
| Liu et al. 2015 (83) | 155.9 ± 34.9 | 2.5 ± 0.7 | 5.6 (1/18) | 0.0 (0/18) | 0.0 (0/18) | 0.0 (0/18) |
| Tai et al. 2016 (101) | 238.2 ± 73.6 | 5.1 ± 1.4 | 4.3 (2/47) | 4.3 (2/47) | 0.0 (0/47) | 2.1 (1/47) |
| Yoo et al. 2012 (107) | 118.3 ± 36.0 (Lob)  145.2 ± 35.5 (TT) | 3.0 ± 1.2 | 0.6 (1/165) | 0.0 (0/165) | 10.9 (18/165) | 0.0 (0/165) |
| **B.** | **Operating time in minutes** | **Hospital stay in days** | **Transient RLN palsy % (no. of patients)** | **Permanent RLN palsy % (no. of patients)** | **Transient hypocalcemia % (no. of patients)** | **Permanent hypocalcemia % (no. of patients)** |
| **BABA-RT** | | | | | | |
| Bae et al. 2016 (19) | 160.9 ± 27.5 (Lob)  192.8 ± 34.8 (TT) | 3.9 ± 27.5 (Lob)  4.3 ± 1.1 (TT) | 2.5 (3/118) | 0.0 (0/118) | 28.0 (33/118) | 1.7 (2/118) |
| Bae et al. 2018 (20) | 198.4 ± 37.9 | 4.1 ± 0.6 | 11.4 (14/123) | 0.8 (1/123) | 31.7 (39/123) | 1.6 (2/123) |
| Chae et al. 2020 (27) | ﻿221.6 ± 50.2 | ﻿3.2 ± 0.8 | 0.0 (0/56) | 0.0 (0/56) | 10.7 (6/56) | 0.0 (0/56) |
| Chai et al. 2017 (28) | 234.8 ± 36.2 (Lob)  301.1 ± 35.7 (TT) | 3.9 ± 1.2 | 4.0 (2/50) | 0.0 (0/50) | 10.8 (4/37) | 0.0 (0/37) |
| Chai et al. 2017 (29) | 165.1 ± 43.9 | 3.2 ± 0.6 | 19.0 (4/21) | 0.0 (0/21) | 19.0 (4/21) | 4.8 (1/21) |
| Cho et al. 2016 (30) | 290.6 ± 74. 4 | 3.6 ± 0.8 | 6.4 (7/109) | 0.92 (1/109) | 33.0 (36/109) | 1.83 (2/109) |
| He et al. 2016 (49) | 118.8 ± 16.5 | 5.1 ±1.4 | 2.0 (1/50) | 0.0 (0/50) | 20.0 (10/50) | 0.0 (0/50) |
| Kim et al. 2018 (61) | 151.5 ± 39.4 | 3.5 ± 0.5 | 2.9 (1/43) | 0.0 (0/43) | 23.5 (4/17) | 0.0 (0/17) |
| Kim et al. 2017 (64) | 184.9 ± 41.8 | 4.3 ± 1.0 | 4.5 (13/289) | 0.7 (2/289) | 38.6 (44/114) | 0.9 (1/114) |
| Kim et al. 2015 (66) | 175.2 ± 3.1 | 3.9 ± 0.5 | 2.6 (8/300) | 0.0 (0/300) | 23.0 (33/143) | 1.3 (2/143) |
| Kim et al. 2011 (67) | 196 ± 45.0 | 3.1 ± 0.7 | 1.4 (1/69) | 0.0 (0/69) | 33.3 (23/69) | 1.4 (1/69) |
| Lee et al. 2013 (72) | 236.3 ± 49.0 (Lob)  287.2 ± 45.2 (TT) | 3.5 ± 1.15 | 3.0 (3/100) | 0.0 (0/100) | 21.6 (19/88) | 0.0 (0/88) |
| Lee et al. 2010 (77) | 206 ± 36 | NR | 16.0 (17/109) | 0.9 (1/109) | 19.2 (21/109) | 1.8 (2/109) |
| Paek et al. 2018 (88) | NR | NR | 4.2 (3/71) | 0.0 (0/71) | 2.8 (2/71) | 0.0 (0/71) |
| Yoo et al. 2012 (107) | 161.0 ± 44.5 (Lob)  189.6 ± 71.2 (TT) | 2.9 ± 1.1 | 0.0 (0/46) | 0.0 (0/46) | 10.8 (5/46) | 0.0 (0/46) |
| **C.** | **Operating time in minutes** | **Hospital stay in days** | **Transient RLN palsy % (no. of patients)** | **Permanent RLN palsy % (no. of patients)** | **Transient hypocalcemia % (no. of patients)** | **Permanent hypocalcemia % (no. of patients)** |
| **MIVAT** | | | | | | |
| Bellotti et al. 2019 (24) | 74 ± 7.2 | NR | 1.8 (2/110) | 0.9 (1/110) | 10.0 (11/110) | 3.6 (4/110) |
| Dedinivitis et al. 2005 (36) | 55 (45-125) | 0.0 ± 0 | 0.0 (0/12) | 0.0 (0/12) | 0.0 (0/12) | 0.0 (0/12) |
| Dobrinja et al. 2009 (37) | 82.6 (35-125) (Lob)  118.7 (63-162) (TT) | 1.9 (1-6) | 4.2 (2/47) | 0.0 (0/47) | 23.4 (11/47) | 0.0 (0/47) |
| Fan et al. 2009 (40) | 35 (20-70) (Lob)  58 (35-90) (TT) | 2.5 (1-5) | 2.3 (7/300) | 1.7 (5/300) | 3.0 (9/300) | NR |
| Fik et al. 2012 (42) | 77 ± 24 (Lob)  108 ± 33 (TT) | NR | 1.7 (1/60) | 1.7 (1/60) | 83 (5/60) | NR |
| Frank et al. 2010 (44) | 86.5 ± 39.3 | NR | 9.6 (56/583) | 0.2 (1/583) | 22.1 (52/234) | 1.3 (3/234) |
| Gagner et al. 2001 (45) | 220 (120-330) | NR | NR | NR | 5.6 (1/18) | NR |
| Hegazy et al. 2007 (50) | 105 (90-180) (Lob)  136 (120-190) (TT) | NR | 3.0 (1/33) | 0.0 (0/33) | 3.0 (1/33) | 0.0 (0/33) |
| Kim et al. 2013 (60) | 113 (Lob)  149 (TT) | 1.4 | 17.0 (9/53) | 1.9 (1/53) | 11.3 (6/53) | 0.0 (0/53) |
| Lai et al. 2008 (69) | 91 (43-131) (Lob)  151 (103-125) (TT) | NR | 2.5 (1/40) | 0.0 (0/40) | 10.0 (2/20) | 0.0 (0/20) |
| Lang et al. 2013 (71) | 60 (23 – 155) | 2 ± 0 | 0.0 (0/13) | 0.0 (0/13) | 7.7 (1/45) | 0.0 (0/45) |
| Materazzi et al. 2014 (84) | 46.5 ± 10.5 | 1.2 (1-3) | 3.3 (1/30) | 0.0 (0/30) | NR | NR |
| Miccoli et al. 2015 (85) | 41 ± 14 | 1.5 | NR | 1.2 (30/2,412) | 5.0 (120/2,412) | 0.4 (10/2,412) |
| Miccoli et al. 2009 (86) | NR | NR | NR | 2.9 (5/170) | NR | 3.5 (6/170) |
| Schabram et al. 2004 (96) | 80 (25-180) | NR | 2.0 (4/196) | 0.5 (1/196) | 5.6 (11/196) | 0.0 (0/196) |
| Shan et al. 2012 (97) | 66.4 ± 17.6 | 2.3 (2-4) | 4.2 (1/24) | 0.0 (0/24) | 4.2 (1/24) | 0.0 (0/24) |
| Ujiki et al. 2006 (103) | 102 ± 4 (Lob)  190 ± 7 (TT) | 1 (0-1) | 4.5 (1/22) | 0.0 (0/22) | NR | 0.0 (0/22) |
| Yu et al. 2012 (108) | 109 ± 49 | 4.7 ± 1.9 | 0.0 (0/24) | 0.0 (0/24) | 0.0 (0/24) | 0.0 (0/24) |

| **Supplementary Table 4. *(continued)*** Surgical and post-surgical outcomes | | | | | | |
| --- | --- | --- | --- | --- | --- | --- |
| **D.** | **Operating time in minutes** | **Hospital stay in days** | **Transient RLN palsy % (no. of patients)** | **Permanent RLN palsy % (no. of patients)** | **Transient hypocalcemia % (no. of patients)** | **Permanent hypocalcemia % (no. of patients)** |
| **TOETVA** | | | | | | |
| Ahn et al. 2020 (12) | 110.3 ± 35.7 | 3.6 ± 1.7 | 4.7 (7/150) | 0.7 (1/150) | 3.3 (5/150) | 1.3 (2/150) |
| Anuwong 2016 (16) | 115.5 (75-300) | 3.6 (2-7) | 3.3 (2/60) | 0.0 (0/60) | 5.0 (3/60) | 5.0 (0/60) |
| Anuwong et al. 2018 (17) | 97 ± 40.50 | 3.2 ± 0.53 | 4.0 (8/200) | 0.0 (0/200) | 17.5 (35/200) | 0.0 (0/200) |
| Bakkar et al. 2017 (21) | 122 (90-150) | NR | 0.0 (0/5) | 0.0 (0/5) | NR | NR |
| Fernandez-Ranvier et al. 2020 (41) | 161.8 ± 42.4 (Lob)  213.4 ± 71.7 (TT)  136.7 ± 109.8 (CT) | 1.6 (0-5) | 3.3 (5/152) | 2.0 (3/152) | 4.7 (7/152) | 0.0 (0/152) |
| Guo et al. 2020 (48) | 172.0 ± 5.3 | 4.3 ± 0.1 | NR | NR | NR | NR |
| Kasemsiri et al. 2020 (59) | 123.8 ± 26.3 | 3.3 ± 0.7 | 12.5 (4/32) | 3.1 (1/32) | NR | NR |
| Park et al. 2019 (89) | 147.5 (100-250) (Lob) | 4.8 (2-10) | 3.1 (2/65) | 1.5 (1/65) | 50.0 (5/10) | 0.0 (0/10) |
| Perez-Soto et al. 2019 (91) | 216.7 ± 62.5 | 2.0 ± 1.4 | 10.0 (2/20) | 0.0 (0/20) | 25.0 (5/20) | 0.0 (0/20) |
| Razavi et al. 2017 (94) | 188 (89-343) | NR | NR | 0.0 (0/20) | NR | NR |
| Tae et al. 2018 (100) | 158.5 ± 18.4 | NR | 7.1 (1/14) | 0.0 (0/14) | 7.1 (1/14) | 0.0 (0/14) |
| Tesseroli et al. 2018 (102) | 196.1 ± 27.8 | 0.8 ± 0.4 | NR | NR | NR | NR |
| Wang et al. 2018 (104) | 124 (Lob, Ist, STT)  172 (Lob + CND)  205 (TT + CND) | 4.1 (3-5) | 0.0 (0/18) | 0.0 (0/18) | 0.0 (0/18) | 0.0 (0/18) |
| Yi et al. 2017 (105) | 152 ± 51.4 | 4.7 ± 3.8 (3-20) | 5.0 (1/20) | 0.0 (0/20) | 15.0 (3/20) | 0.0 (0/20) |
| **E.** | **Operating time in minutes** | **Hospital stay in days** | **Transient RLN palsy % (no. of patients)** | **Permanent RLN palsy % (no. of patients)** | **Transient hypocalcemia % (no. of patients)** | **Permanent hypocalcemia % (no. of patients)** |
| **RA-ET** | | | | | | |
| Alshehri et al. 2017 (15) | 123.4 ± 23.4 | NR | NR | NR | NR | NR |
| Ban et al. 2016 (23) | 143 ± 29.7 | 5.2 ± 0.8 | 0.0 (0/8) | 0.0 (0/8) | 0.0 (0/8) | 0.0 (0/8) |
| Byeon et al. 2016 (25) | 131 ± 50.5 | 5.9 ± 1.1 | 11.1 (2/18) | 0.0 (0/18) | 5.6 (1/18) | 0.0 (0/18) |
| Chung et al. 2015 (33) | 152 ± 48 | 2.5 | 2.1 (1/47) | 0.0 (0/47) | 0.0 (0/47) | 0.0 (0/47) |
| Park et al. 2015 (90) | 140 (120-180) | 3.8 ± 0.7 | 0.0 (0/11) | 0.0 (0/11) | 0.0 (0/11) | 0.0 (0/11) |
| **F.** | **Operating time in minutes** | **Hospital stay in days** | **Transient RLN palsy % (no. of patients)** | **Permanent RLN palsy % (no. of patients)** | **Transient hypocalcemia % (no. of patients)** | **Permanent hypocalcemia % (no. of patients)** |
| **RA-RT** | | | | | | |
| Alshehri et al. 2017 (15) | 167.10 ± 40.5 | NR | NR | NR | NR | NR |
| Sung et al. 2016 (99) | 191 ± 143 | NR | 5.0 (1/20) | 0.0 (0/20) | 33.3 (1/3) | 0.0 (0/3) |
| **G.** | **Operating time in minutes** | **Hospital stay in days** | **Transient RLN palsy % (no. of patients)** | **Permanent RLN palsy % (no. of patients)** | **Transient hypocalcemia % (no. of patients)** | **Permanent hypocalcemia % (no. of patients)** |
| **GTET** | | | | | | |
| Jantharapattana et al. 2017 (2) | ﻿297.5 ± 56.2 | ﻿2.2 ± 0.9 | NR | 6.3 (1/16) | NR | NR |
| Cabot et al. 2012 (26) | 184.9 ± 26.0 | 5.0 ± 0.8 | 0.0 (0/30) | 0.0 (0/30) | 33.3 (5/15) | 0.0 (0/15) |
| Duncan et al. 2007 (38) | 138.5 (68-322) | NR | 6.3 (2/32) | 0.0 (0/32) | 0.0 (0/32) | 0.0 (0/32) |
| Duncan et al. 2009 (39) | 147 (63-331) | NR | 3.8 (2/53) | 0.0 (0/53) | 0.0 (0/53) | 0.0 (0/53) |
| Jeong et al. 2009 (53) | 138.5 ± 49.0 | 3.4 ± 0.9 | 5.5 (8/275) | 0.4 (1/275) | 31.0 (11/35) | 0.0 (0/35) |
| Kang et al. 2009 (56) | 129.4 ± 51.3 (B)  135.5 ± 47 (M) | 3.3 ± 1.7 (B)  3.4 ± 0.9 (M) | 2.2 (13/581) | 0.3 (2/581) | 3.3 (19/581) | 0.0 (0/581) |
| Kim et al. 2017 (62) | 142.6 ± 3.3 | NR | 15.0 (17/200) | 0.5 (1/200) | 22.4 (45/200) | 2.7 (3/200) |
| Lang et al. 2013 (71) | 112 (50-245) | 2.6 ± 0.8 | 6.3 (6/96) | 1.6 (2/96) | 3.2 (1/31) | 0.0 (0/31) |
| Lee et al. 2011 (74) | 142.7 ± 52.1 | 3.2 ± 1.9 | 3.1 (3/96) | 1.0 (1/96) | 0.0 (0/2) | 0.0 (0/2) |
| Lee et al. 2011 (82) | 127.1 ± 40.8 (STT)  158.4 ± 50.5 (TT) | 3.4 ± 2.8 | 5.1 (29/570) | 0.2 (1/570) | 19.7 (14/71) | 1.4 (1/71) |
| Lee et al. 2013 (79) | 193.3 ± 52.5 | 6.5 ± 2.4 | 5.9 (5/84) | 2.4 (2/84) | NR | NR |

Operating time and hospital stay are reported as mean ± SD or median (range); *RLN* recurrent laryngeal nerve; *Lob* lobectomy; *STT* subtotal thyroidectomy; *TT* total thyroidectomy; *CT* completion thyroidectomy; *CND* central neck dissection; *B* benign; *M* malignant; *SP* single port, *DP* double port; * early group; ** late group; ° discharged within one day; *NR* not reported

| **Supplementary Table 4. *(continued)*** Surgical and post-surgical outcomes | | | | | | |
| --- | --- | --- | --- | --- | --- | --- |
| **H.** | **Operating time in minutes** | **Hospital stay in days** | **Transient RLN palsy % (no. of patients)** | **Permanent RLN palsy % (no. of patients)** | **Transient hypocalcemia % (no. of patients)** | **Permanent hypocalcemia % (no. of patients)** |
| **RATS** | | | | | | |
| Aliyev et al. 2012 (13) | 183 ± 11 | 1.1 ± 0.1 | NR | NR | 12.0 (2/16) | NR |
| Arora et al. 2016 (18) | 228 | 0 ± 0 | 6.3 (1/16) | 0.0 (0/16) | NR | NR |
| Ban et al. 2013 (22) | 124.8 ± 32.5 | 3.3 ± 1.7 | 1.2 (37/3,000) | 0.3 (8/3,000) | 37.4 (408/1,090) | 1.1 (12/1,090) |
| Cabot et al. 2012 (26) | 165.7 ± 29.4 | 5.1 ± 0.7 | 0.0 (0/30) | 0.0 (0/30) | 33.3 (5/15) | 0.0 (0/15) |
| Ciabatti et al. 2012 (35) | 178.5 ± 24.2 | NR | 6.9 (2/29) | 0.0 (0/29) | 0.0 (0/29) | 0.0 (0/29) |
| Foley et al. 2010 (43) | 232 ± 65.7 | NR | 0.0 (0/11) | NR | 9.1 (1/11) | 0.0 (0/11) |
| Garstka et al. 2018 (46) | 162.7 ± 72.6 | 0.6 ± 0.9 | 2.9 (1/35) | 0.0 (0/35) | 0.0 (0/35) | 0.0 (0/35) |
| Giulianotti et al. 2012 (47) | 177.4 ± 40 | 1.1 (1-1.4) | 10.0 (1/10) | 0.0 (0/10) | 0.0 (0/10) | 0.0 (0/10) |
| Kandil et al. 2012 (54) | 131 (101-203) | 1 | 0.0 (0/10) | 0.0 (0/10) | 0.0 (0/10) | 0.0 (0/10) |
| Kandil et al. 2012 (55) | 113.5 ± 63.6 | NR | 7.0 (7/100) | 0.0 (0/100) | 10.0 (10/100) | 0.0 (0/100) |
| Kang et al. 2009 (57) | 144.0 ± 43.5 | 3.3 ± 0.8 | 4.0 (13/338) | 1.0 (3/338) | 41.0 (43/105) | 0.0 (0/105) |
| Kang et al. 2011 (58) | 136.7 ± 44.4 | 3.0 ± 0.5 | 4.2 (42/1,000) | 0.3 (3/1,000) | 38.8 (145/373) | 0.0 (0/373) |
| Kim et al. 2018 (65) | 134.5 ± 122 | 3.3 ± 3.0 | 2.5 (126/5,000) | 0.4 (18/5,000) | 48.1 (844/1,837) | 1.3 (25/1,837) |
| Kuppersmith et al. 2010 (68) | 196* & 109**  (48-270) (Lob)  278* & 168** (132-328) (TT) | 30/31° | NR | NR | 0.0 (0/31) | 0.0 (0/31) |
| Landry et al. 2011 (70) | 121 (74-199) | 23 (<23h)  2 (48h) | 20.0 (5/25) | 0.0 (0/25) | NR | NR |
| Lee et al. 2011 (73) | 119.7 ± 61.8 | 3.4 ± 2.3 | 3.0 (60/2,014) | 0.4 (9/2,014) | 34.7 (257/740) | 0.05 (1/740) |
| Lee et al. 2011 (74) | 110.1 ± 50.7 | 2.8 ± 1.1 | 1.8 (3/163) | 0.6 (1/163) | 12.5 (6/48) | 0.0 (0/48) |
| Lee et al. 2012 (75) | NR | NR | 20.5 (18/88) | NR | NR | NR |
| Lee et al. 2011 (76) | 132.4 ± 48.5 | 2.9 ± 0.8 | 4.3 (45/1,043) | 0.0 (0/1,043) | 18.4 (192/1,043) | 0.0 (0/1043) |
| Lee et al. 2014 (79) | NR | 3.4 ± 1.5 | 5.0 (3/60) | NR | 42.0 (25/60) | 0.0 (0/60) |
| Lee et al. 2013 (80) | NR | NR | 2.3 (1/43) | NR | 46.5 (20/43) | 0.0 (0/43) |
| Lee et al. 2011 (81) | 126.8 ± 34.5 (STT)  151.7 ± 35.4 (TT) | 3.3 ± 0.8 | 3.3 (19/580) | 0.7 (4/580) | 37.8 (51/135) | 0.0 (0/135) |
| Lee et al. 2013 (82) | 132.8 ± 28.1 (STT) 179.1 ± 36.4 (TT) | 3.6 | 0.0 (0/400) | 1.0 (4/400) | 51.7 (75/145) | 1.4 (2/145) |
| Materazzi et al. 2014 (84) | 121.5 ± 46.8 | 1.9 (1-4) | 3.1 (1/32) | 0.0 (0/32) | NR | NR |
| Noureldine et al. 2013 (87) | 163.3 ± 78.6 | 0.5 ± 0.5 | 4.1 (1/24) | 0.0 (0/24) | 8.3 (2/24) | 0.0 (0/24) |
| Piccoli et al. 2019 (92) | 117.5 ± 40.3 | 1.8 ± 0.8 | 2.6 (12/449) | 0.2 (1/449) | 16.0 (72/449) | 0.0 (0/449) |
| Prete et al. 2019 (93) | 190.5 (75-377) | 2.3 | 0.0 (0/12) | 0.0 (0/12) | 50.0 (1/2) | 0.0 (0/2) |
| Ryu et al. 2010 (95) | 114.9 ± 27 (SP)  135.0 ± 35.9 (DP) | 3.1 ± 0.6 (SP)  3.4 ± 3.1 (DP) | 3.8 (40/1,047) | 0.3 (3/1,047) | 35.8 (133/371) | 0.0 (0/371) |
| Stang et al. 2018 (98) | 81 ± 26 (Lob)  109 ± 29 (TT) | 116/141°(Lob)  84/160° (TT) | 6.0 (18/301) | 1.3 (4/301) | 8.2 (15/182) | 1.1 (2/182) |
| Yi et al. 2013 (106) | NR | NR | NR | 1.0 (1/98) | 53.1 (52/98) | 3.1 (3/98) |

Operating time and hospital stay are reported as mean ± SD or median (range); *RLN* recurrent laryngeal nerve; *Lob* lobectomy; *STT* subtotal thyroidectomy; *TT* total thyroidectomy; *CT* completion thyroidectomy; *CND* central neck dissection; *B* benign; *M* malignant; *SP* single port, *DP* double port; * early group; ** late group; ° discharged within one day; *NR* not reported
